# A genetically encoded single-wavelength sensor for imaging cytosolic and cell surface ATP

**DOI:** 10.1038/s41467-019-08441-5

**Published:** 2019-02-12

**Authors:** Mark A. Lobas, Rongkun Tao, Jun Nagai, Mira T. Kronschläger, Philip M. Borden, Jonathan S. Marvin, Loren L. Looger, Baljit S. Khakh

**Affiliations:** 1 Department of Physiology, David Geffen School of Medicine, University of California Los Angeles, Los Angeles, CA 90095-1751 USA; 2 Janelia Research Campus, 19700 Helix Drive, Ashburn, VA 20147 USA; 3 Department of Neurobiology, David Geffen School of Medicine, University of California Los Angeles, Los Angeles, CA 90095-1751 USA; 4 Present Address: Koniku Inc., 740 Heinz Avenue, Berkeley, CA 94710 USA; 5 Present Address: Department of Neurophysiology, Center for Brain Research, Medical University of Vienna, Spitalgasse 4, 1090 Vienna, Austria

## Abstract

Adenosine 5′ triphosphate (ATP) is a universal intracellular energy source and an evolutionarily ancient, ubiquitous extracellular signal in diverse species. Here, we report the generation and characterization of single-wavelength genetically encoded fluorescent sensors (iATPSnFRs) for imaging extracellular and cytosolic ATP from insertion of circularly permuted superfolder GFP into the epsilon subunit of F_0_F_1_-ATPase from *Bacillus PS3*. On the cell surface and within the cytosol, iATPSnFR^1.0^ responds to relevant ATP concentrations (30 μM to 3 mM) with fast increases in fluorescence. iATPSnFRs can be genetically targeted to specific cell types and sub-cellular compartments, imaged with standard light microscopes, do not respond to other nucleotides and nucleosides, and when fused with a red fluorescent protein function as ratiometric indicators. After careful consideration of their modest pH sensitivity, iATPSnFRs represent promising reagents for imaging ATP in the extracellular space and within cells during a variety of settings, and for further application-specific refinements.

## Abstract (web summary)

ATP has essential roles in cell signalling and energy homeostasis and biosensors to detect it have many potential applications. Here, the authors develop a new ATP sensor that can be targeted to the membrane or cytosol.

## Introduction

Given widespread roles in energy homeostasis and cell signaling^1–5^, several methods have been used to detect ATP using small-molecule chemical approaches^6^, firefly luciferase^7,8^, ion channel-expressing “sniffer” cells^9–11^, voltammetry^12^, and different types of microelectrodes^13,14^. Although progress has been made, these methods, however, lack spatial resolution, cannot be easily used in tissue slices or in vivo, respond to off-target ligands, need sensitive photon-counting cameras, are imprecise, and/or unavoidably damage tissue during probe placement. Importantly, some of these methods cannot be genetically targeted to specific cells, sub-cellular compartments or whole organisms, and they all lack cellular-scale spatial resolution. Although luciferase is genetically encoded^15^, it requires the exogenous substrate luciferin, addition of which complicates use in tissue slices and in vivo. It also saturates at nanomolar ATP, lower than concentrations expected during ATP signaling (~1 μM to ~1 mM) and therefore may most reliably respond as a step function indicator. Additionally, luciferase’s bioluminescent output yields low photon fluxes and limits cellular-scale resolution imaging. Despite these potential limitations, several pioneering studies have successfully used genetically targeted luciferase-based probes to image ATP dynamics and this remains a useful approach in the purinergic signaling and intracellular ATP homeostasis fields^16–19^. However, to address the aforementioned limitations more generally, genetically-encoded fluorescent ATP sensors have been developed, including Perceval and PercevalHR^20,21^, ATeam^22^, and QUEEN^23^. These sensors are valuable, but they also have some limitations. Specifically, they either respond to ADP/ATP ratio instead of ATP concentration or are susceptible to optical overlap with cellular sources of auto-fluorescence, and some are incompatible with single-wavelength fluorescence imaging, the workhorse of functional fluorescence microscopy^24^.

In the present study, our goals were to (1) develop an ATP sensor that could be used in routine fluorescence imaging, e.g., at GFP’s 488 nm excitation and ~525 nm emission and (2) deploy such a sensor on the cell surface and within cells to image ATP. QUEEN is an excitation ratio sensor and ATeam is a fluorescence resonance energy transfer (FRET) sensor—these methods require customized equipment, substantially slow down imaging rates, complicate use with other reagents, and often have a lower signal-to-noise ratio (SNR) than single fluorescence-wavelength intensity indicators. Furthermore, although FRET-based indicators can be used in single-wavelength mode by exciting at the donor’s excitation peak and measuring the acceptor’s sensitized emission, such strategies fail to benefit from the ratiometric nature of FRET and are problematic to quantify with single-wavelength imaging^25^. Existing FRET-based ATP sensors also employ CFP/YFP FRET, which would require excitation at 442 nm for imaging YFP emission: 442 nm lasers are not standard on confocal microscopes and CFP in the existing sensors has one of the lowest quantum yields of the available GFP-based proteins. This would translate to inefficient energy transfer from the donor’s excited state to the acceptor by dipole–dipole coupling. In contrast, our focus to develop a GFP-based ATP sensor is based on advances made with the generation of GFP-based glutamate sensors, which are a reliable workhorse in a variety of applications^26,27^. Furthermore, GFP-based sensors have recently been made for a variety of small molecules such as dopamine, acetylcholine, and GABA^28–31^. Our approach of using GFP does not of course vitiate other aforementioned approaches, such as the continued use of wild type or modified luciferases^16–19,32^. From these perspectives, our approach is complementary and we believe more readily integrated into standard imaging regimes for cellular-scale imaging in a variety of biological applications in cells and in vivo. As such, we report a GFP-based genetically encoded single-wavelength sensor for imaging cytosolic and cell surface ATP.

## Results

### Generation of single-wavelength ATP sensors

**Fig. 1:**
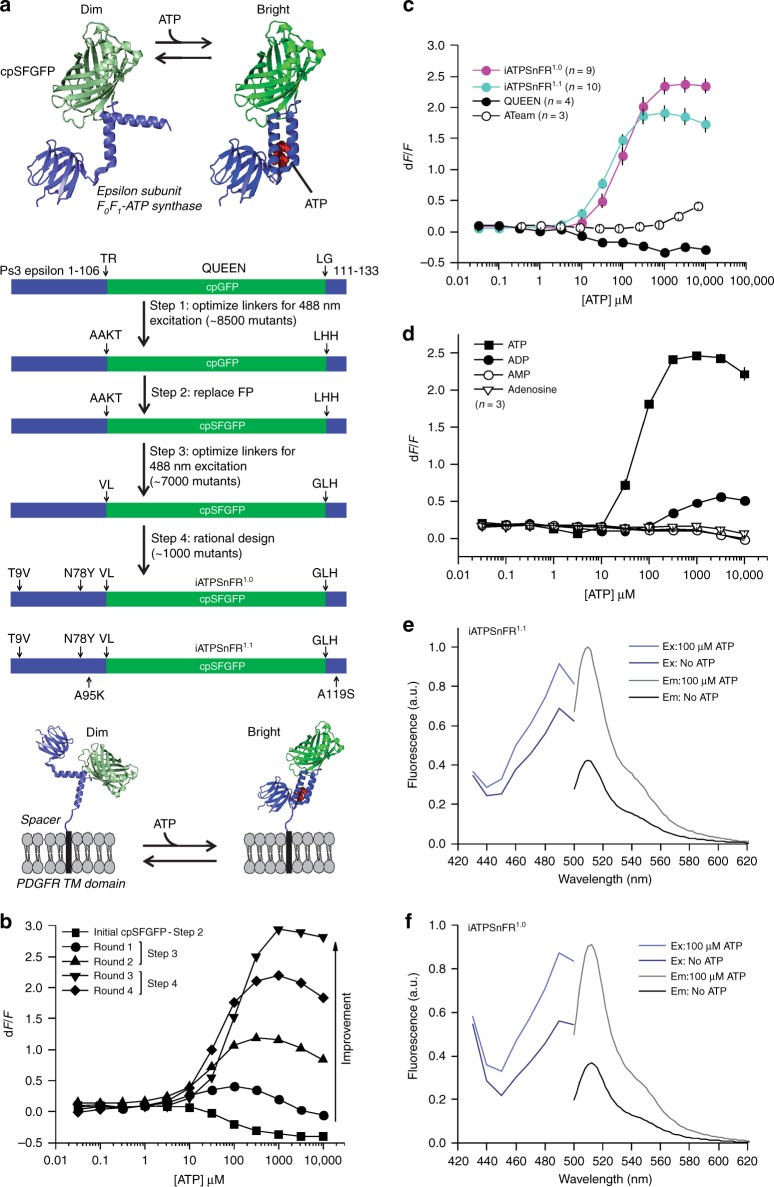
Design and optimization of a single-wavelength ATP sensor. **a** Schematic showing the design and workflow used to optimize QUEEN-7µ into a single-wavelength ATP sensor with the goal of displaying the sensor on the surface of cells. **b** Dose–response curves of iATPSnFR over several successive rounds of mutagenesis (Ex: 488 nm, Em: 515 nm). Fluorescence quenching at very high ATP concentrations can be observed in addition to binding-dependent increases. **c** Dose–response curves for purified ATeam, QUEEN-7µ, iATPSnFR^1.0^, and iATPSnFR^1.1^. ATeam dose–response curves were acquired with Ex: 435 nm and Em: 530 nm. The other constructs were with Ex: 488 nm, Em: 515 nm. **d** Dose–response curves of purified iATPSnFR^1.1^ to ATP, ADP, AMP, and adenosine. **e**, **f** Excitation and emission spectra for iATPSnFR^1.0^ and iATPSnFR^1.1^ in solution (the traces are the average from 48 replicates each in a 96-well plate). The error bars represent the s.e.m. and in some cases are smaller than the symbols used for the mean. When greater than one (in the case of exemplar traces and graphs), *n* is provided in the figure panels and refers to the number of independent evaluations

We considered and initially explored a variety of potential methods to generate an ATP sensor. After unsuccessful attempts to modify ATP-gated P2X receptors^33^ and the bacterial regulatory protein GlnK1 (as in Perceval^20^) to suit our needs, we turned to microbial F_0_F_1_-ATP synthase epsilon subunits^34^, which have been adapted to create the FRET sensor ATeam^22^ and the excitation ratio sensor QUEEN^22,23^, upon which our sensors are based. The epsilon subunit is predicted to undergo a large conformational change^34^ upon binding ATP (Fig. 1a), and the homologue from the thermophilic bacterium *Bacillus PS3* displays appropriate ATP-binding sensitivity^35^. The epsilon subunit comprises eight N-terminal β-strands followed by two C-terminal α-helices (Fig. 1a), which extend away from the β-strands in the absence of ATP, but upon binding cradle ATP up against the β-strands.

Circularly permuted (cpGFP)^36^ was inserted between the two α-helices of the epsilon subunit after residue 107 with the expectation that the epsilon subunit conformational change might alter fluorescence. The first linker (L1) initially comprised Thr–Arg, with the second linker (L2) Leu–Gly (Fig. 1a). Based on our past experience with the glutamate sensor iGluSnFR^26,27^, we began mutating residues in the linkers and ~8500 colonies were screened to develop sensors with large ATP-dependent fluorescence intensity increases (d*F*/*F*). The best variant from this screen had a large change in fluorescence (maximum d*F*/*F* of ~3.9). However, it failed to express on the surface of HEK293 cells when cloned into the pDisplay mammalian expression vector, which uses an IgG secretion signal and a platelet-derived growth factor receptor (PDGFR) transmembrane domain to anchor it to the membrane. We reasoned that a more stable form of GFP might improve folding and trafficking, and thus cloned circularly permuted superfolder GFP^36^ (cpSFGFP) in place of cpGFP. Replacing cpGFP with cpSFGFP remedied the surface trafficking in HEK293 cells (see later section), but greatly diminished ATP-evoked changes in fluorescence. To correct this, we re-optimized L1 and L2 for the cpSFGFP construct by mutating amino acids in the linkers and slightly changing their length; ~7000 colonies were screened (Fig. 1a, b). We also mutated amino acids (Thr9Val and Asn78Tyr) predicted from molecular modeling to decrease dimer formation. Through this process, we developed two sensors that displayed large ATP-dependent increases in fluorescence (Fig. 1a, b). In the sensor we termed iATPSnFR^1.0^, the L1 linker was changed from Thr–Arg to Val–Leu, and L2 from Leu–Gly to Gly–Leu–His. We developed a second sensor (iATPSnFR^1.1^) with improved sensitivity by mutating amino acids near the ATP-binding pocket. iATPSnFR^1.1^ differs from iATPSnFR^1.0^ by two mutations (Ala95Lys and Ala119Ser; Fig. 1a; Supplementary Figure 1). Both iATPSnFR^1.0^ and iATPSnFR^1.1^ show marked improvement over QUEEN-7µ, which does not function as a single-wavelength sensor, and over ATeam for the same ATP concentration range (Fig. 1c). Furthermore, inserting cpSFGFP into Queen did not result in a sensor with ATP-evoked fluorescence increases. Purified iATPSnFR^1.0^ had a maximum d*F*/*F* of ~2.4 and an EC_50_ of ~120 µM, whereas purified iATPSnFR^1.1^ had a maximum d*F*/*F* of ~1.9 and an EC_50_ of ~50 µM (Fig. 1c). Purified iATPSnFRs were not sensitive to ADP, AMP, or adenosine at concentrations equivalent to ATP (Fig. 1d). Both proteins displayed similar fluorescence spectra (peak excitation 490 nm, peak emission 512 nm; Fig. 1e, f). In the presence of ATP, an increase in peak excitation and emission was observed. Supplementary Figure 2 shows that the fluorescence peak at 400 nm, which is seen in many GFP-based sensors including QUEEN, was essentially ATP-independent. Thus, our bacterial-based screen yielded a genetically encoded ATP sensor with micromolar sensitivity, large 488 nm-excited emission intensity d*F*/*F*, and little sensitivity to ATP metabolites (Fig. 1).

### Testing of ATP sensors in HEK293 cells

**Fig. 2:**
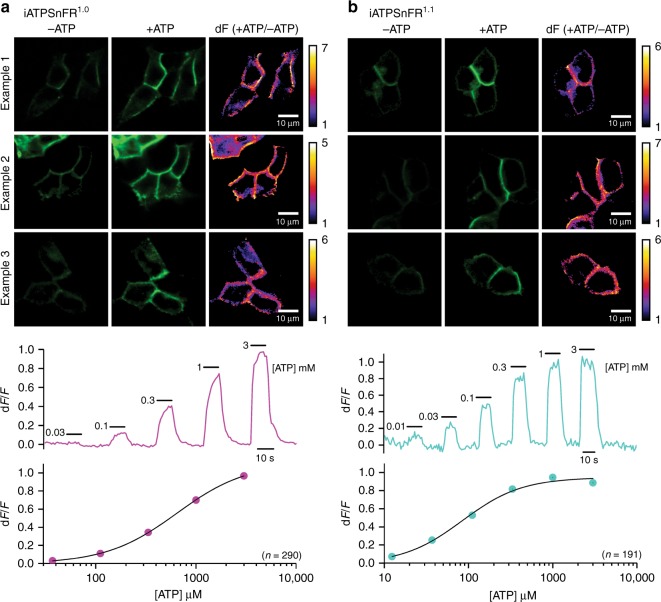
Representative images and traces for responses measured with cell surface iATPSnFRs. **a** The upper panels show three examples of ATP responses for HEK293 cells expressing iATPSnFR^1.0^. The lower panels show representative traces and an average dose–response curve from 290 cells. **b** As in (**a**), but for iATPSnFR^1.1^. The dose–response curve represents an average from 191 cells. In the dose–response curves, the error bars represent the s.e.m. and in some cases are smaller than the symbols used for the mean. *n* numbers are provided in the figure panels and refer to the number of cells

**Fig. 3:**
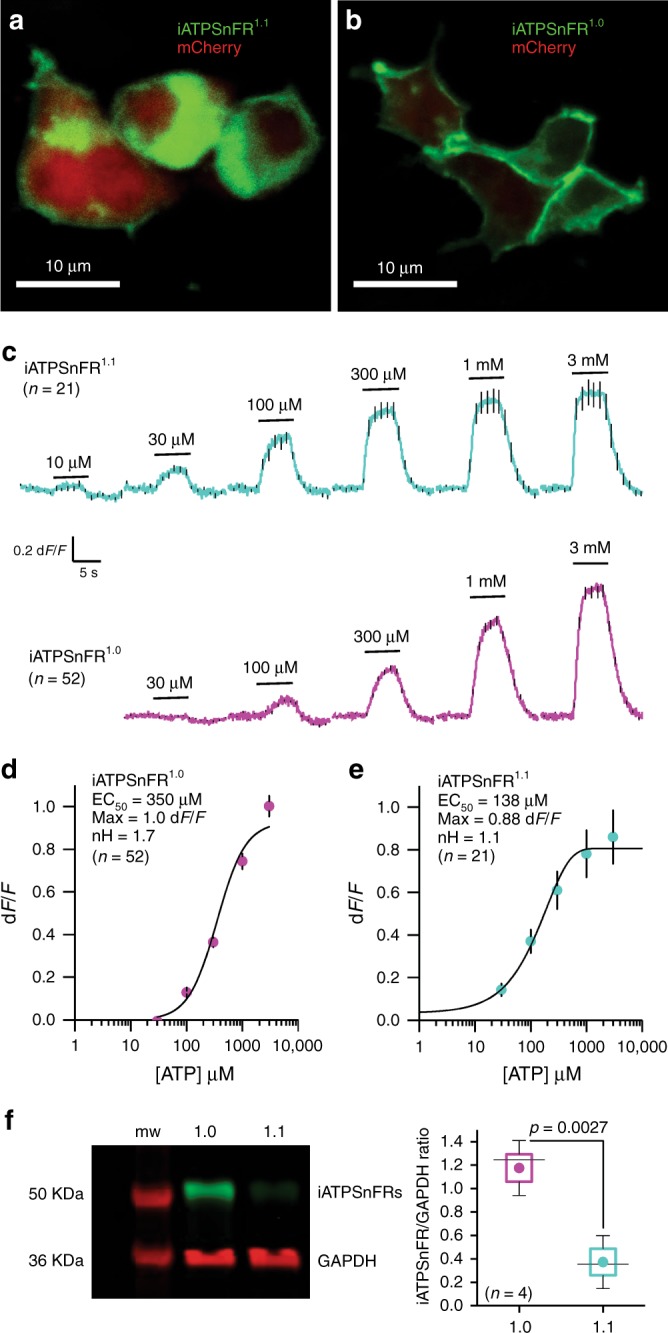
Characterization of cell surface iATPSnFRs in HEK293 cells. **a** Single planes from a confocal z-stack of transiently transfected HEK293 cells expressing cytoplasmic mCherry and membrane-displayed iATPSnFR^1.0^. **b** As in (**a**), but for iATPSnFR^1.1^ transiently expressed in HEK293 cells. **c** Average traces for iATPSnFR^1.1^ (green) and iATPSnFR^1.0^ (red) over a range of concentrations from 10 µM to 3 mM measured with wide field epifluorescence imaging. **d**, **e** Dose–response curves for iATPSnFR^1.0^ and iATPSnFR^1.1^ displayed on the surface of HEK293 cells. Sensitivity (EC_50_), total dynamic range (Max) and cooperativity (nH) shown. **f** Representative Western blot from HEK293 cells transfected with iATPSnFR^1.0^ or iATPSnFR^1.1^ probed for GFP (iATPSnFRs) and GAPDH (loading control). The full-uncropped gel is shown in Supplementary Figure 9. In panel (**f**), the graph shows the quantification of iATPSnFR expression from four Western blots normalized to GAPDH loading control. The circle represents the mean, the box the s.e.m., the whiskers the s.d., and the horizontal line the median. The data were compared with an un-paired Students *t* test. In the other panels, the error bars represent the s.e.m. and in some cases are smaller than the symbols used for the mean. *n* numbers are provided in the figure panels and refer to the number of cells for panels (**c** and **d**) and independent evaluations for panel (**f**)

We initially tested the sensors in HEK293 cells with confocal imaging. Figure 2a, b shows three examples for cell surface targeted iATPSnFR^1.0^ and iATPSnFR^1.1^ transiently expressed in HEK293 cells for 24 h. Images are shown before and during ATP applications (1 mM). The ratio of the +ATP and −ATP image is also shown on the right for each image series (d*F*). From these images, it was clear that both iATPSnFRs were expressed in HEK293 cells, targeted to the surface of the cells and that both responded with visibly discernible increases in fluorescence that localized to the cell edges (Fig. 2a, b; representative of 290 and 191 cells for iATPSnFR^1.0^ and iATPSnFR^1.1^, respectively, from four separate transfections each). Supplementary Movies 1 and 2 for iATPSnFR^1.0^ and iATPSnFR^1.1^ also illustrate this point. The traces below each set of images in Fig. 1 show representative traces for the various ATP concentrations shown and the graphs show dose–response relations. From such experiments, we concluded that both iATPSnFR^1.0^ and iATPSnFR^1.1^ functioned as ATP sensors when extracellularly displayed on the plasma membrane. Furthermore, in HEK293 cells co-transfected with cytoplasmic mCherry and membrane-displayed iATPSnFR^1.0^ or iATPSnFR^1.1^ (Fig. 3a), there was clear iATPSnFR fluorescence at the cell edges. In contrast, fluorescence was mostly absent from the cytoplasm, indicating proper membrane trafficking (Fig. 3a, b).

Without optical sectioning and using wide field epifluorescence imaging, both iATPSnFR^1.0^ and iATPSnFR^1.1^ displayed concentration-dependent increases in fluorescence that were rapid, maintained in ATP, and that returned to baseline upon ATP washout (Fig. 3c). iATPSnFR^1.0^ displayed an EC_50_ of ~350 µM with a maximum d*F*/*F* of ~1.0 (Fig. 3d). Similarly, iATPSnFR^1.1^ displayed an EC_50_ of ~140 µM with a maximum d*F*/*F* of ~0.9 (Fig. 3e). Thus, there was a ~3-fold loss of ATP sensitivity and decrease in peak response in both sensors when they were extracellularly displayed compared to the soluble proteins (similar effects are seen with other membrane-displayed sensors^26,27,31^). During these experiments, we noticed that cells expressing iATPSnFR^1.1^ were dimmer than those expressing iATPSnFR^1.0^, and we found lower, but measurable expression levels of iATPSnFR^1.1^ in relation to iATPSnFR^1.0^ (Fig. 3f). However, both were expressed. Thus, iATPSnFR^1.1^ has a lower ATP EC_50_ (more sensitive), but is not as well expressed as iATPSnFR^1.0^. iATPSnFR^1.1^ also showed slightly more intracellular accumulation than iATPSnFR^1.0^ (Fig. 3a).

### Kinetics and ligand specificity of the ATP sensors

**Fig. 4:**
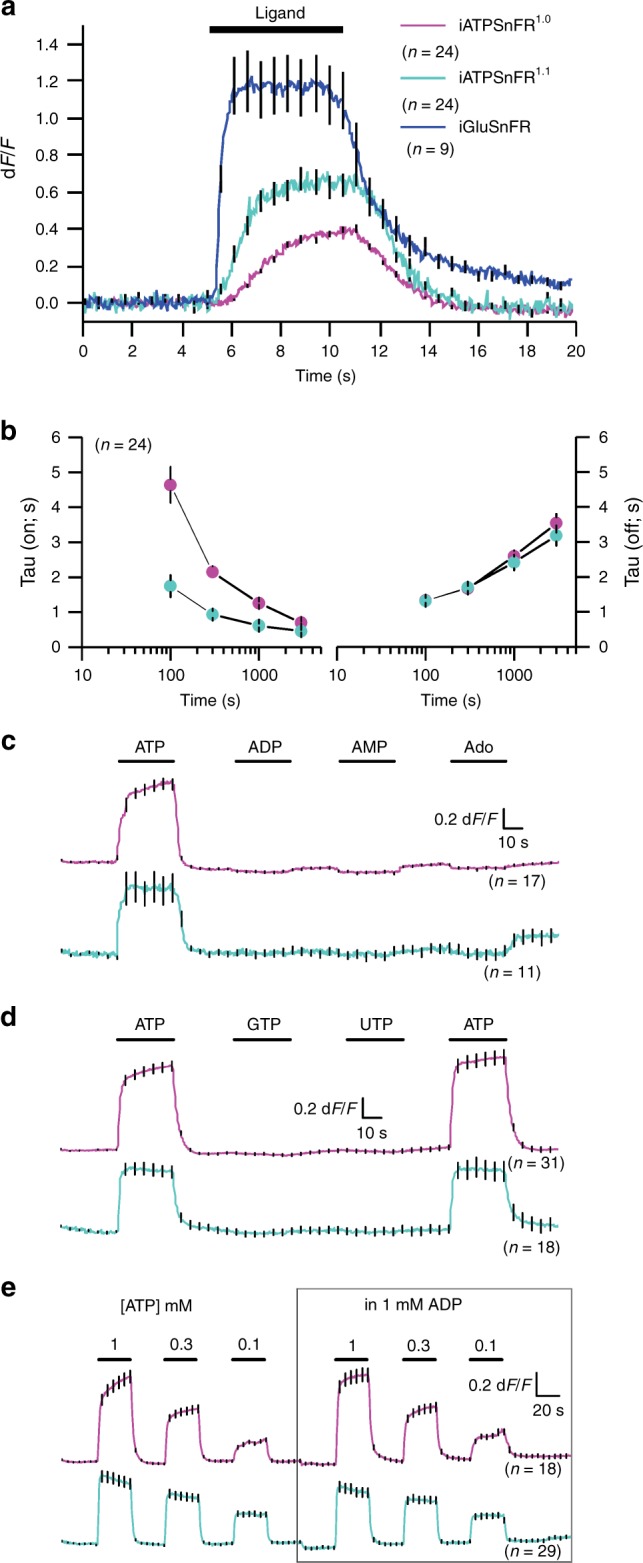
Assessment of the kinetics and ligand selectivity of cell surface ATP sensors. **a** Traces for cell-displayed iATPSnFR^1.0^, iATPSnFR^1.1^, and iGluSnFR kinetics from fast-solution change experiments. We could change solutions in under ~10 ms. **b** Shows the *τ*_on_ and *τ*_off_ for iATPSnFR^1.0^ and iATPSnFR^1.1^ at various ATP concentrations. **c** Shows the response of iATPSnFR^1.0^ and iATPSnFR^1.1^ to ATP, ADP, AMP, and adenosine (1 mM applications). **d** The response of iATPSnFR^1.0^ and iATPSnFR^1.1^ to ATP, GTP, and UTP (1 mM applications). **e** Traces of iATPSnFR^1.0^ and iATPSnFR^1.1^ with three different concentrations of ATP (1, 0.3, and 0.1 mM) with and without 1 mM ADP. ADP has little effect on iATPSnFR responses. The error bars represent the s.e.m. and in some cases are smaller than the symbols used for the mean. *n* numbers are provided in the figure panels and refer to the number of cells

Using a fast solution switcher, we assessed the response kinetics of cell-displayed iATPSnFR^1.0^ and iATPSnFR^1.1^ in relation to iGluSnFR, which displays fast on- and off-rates^26,37^. The time constants for fluorescence increases (*τ*_on_) were dose-dependent and significantly faster for iATPSnFR^1.1^ than iATPSnFR^1.0^ (Fig. 4a, b). In contrast, the time constants for the return to baseline (*τ*_off_) of the ATP-dependent fluorescence increase were not different between the two constructs, implying that the higher equilibrium EC_50_ of iATPSnFR^1.0^ was due to slower association of ATP with the sensor or due to slower subsequent coupling of ATP binding to fluorescence change. In comparison, iGluSnFR was significantly faster than both iATPSnFRs (Fig. 4a, b).

ATP is sequentially degraded to ADP, AMP, and adenosine by ATPases and other ATP-utilizing enzymes in the cytosol and on the cell surface. In accordance with the bacterial lysate data (Fig. 1d), cell surface iATPSnFRs were not sensitive to ADP, AMP, or adenosine at 1 mM (Fig. 4c). Furthermore, iATPSnFRs were not sensitive to 1 mM GTP or 1 mM UTP (Fig. 4d), other nucleoside triphosphates found within the cytosol and in the extracellular space. Since the extracellular space is expected to contain a mixture of ATP and ADP, we evaluated if 1 mM ADP affected the response to 0.1, 0.3, and 1 mM ATP for iATPSnFR^1.0^ and iATPSnFR^1.1^. It did not (Fig. 4e). Thus, iATPSnFRs sense ATP with negligible responses to degradative products or to common competing nucleotides and nucleosides.

### Expression of iATPSnFR^1.0^ in astrocytes and neurons

**Fig. 5:**
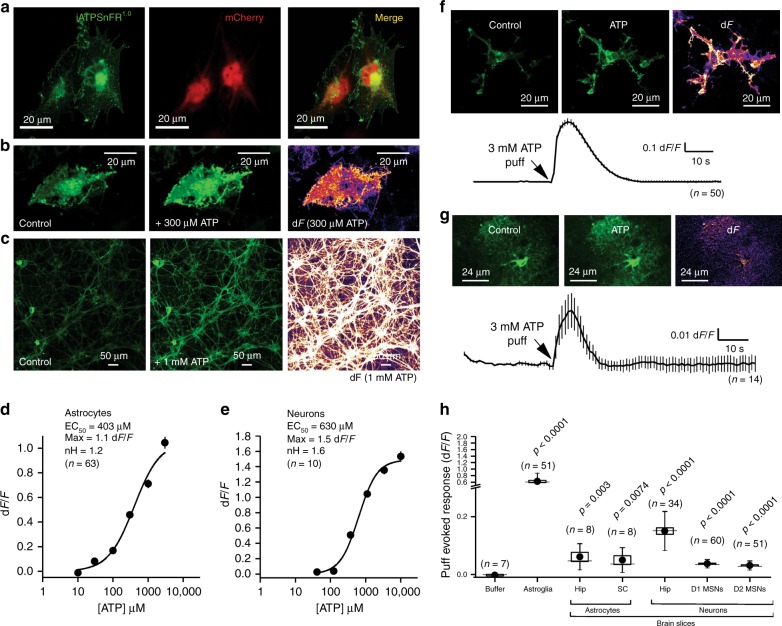
Characterization of cell surface and cytosolic iATPSnFR^1.0^ in astrocytes and neurons. **a** A single plane from a confocal z-stack of a U373MG cell expressing cytoplasmic mCherry and membrane-displayed iATPSnFR^1.0^. **b** Confocal images of U373MG cells expressing iATPSnFR^1.0^ before ATP application (control) and during a 300 µM ATP application (+ATP), as well as the change in fluorescence (d*F*). **c** Confocal images of hippocampal neurons before ATP application (control) and during 1 mM ATP application, as well as the change in fluorescence (d*F*). **d**, **e** Dose–response curves for iATPSnFR^1.0^ when displayed on the surface of U373MG astroglia and hippocampal neurons. **f** A confocal image of a U373MG astroglia cell transfected with iATPSnFR^1.0^ before a 3 mM puff of ATP (control), during the puff (ATP), as well as the change in fluorescence (d*F*). An average trace from all cells with the s.e.m. is also shown. **g** Representative hippocampal astrocyte in acute brain slice expressing iATPSnFR^1.0^ before the application of ATP (control), during the application of 3 mM ATP (ATP), and the dF. An average trace from all cells with the s.e.m. is also shown. **h** Scatter graph summary of the effect of 3 mM ATP on iATPSnFR^1.0^ expressed in the cell types indicated. In panel (**h**), the circle represents the mean, the box the s.e.m., the whiskers the s.d., and the horizontal line the median. *p* Values are shown for statistical comparisons to the buffer puff control using un-paired Student’s *t* tests. Abbreviations: hip hippocampus, SC spinal cord, MSNs medium spiny neurons. Note broken *y*-axis. In other panels, the error bars represent the s.e.m. and in some cases are smaller than the symbols used for the mean. *n* numbers are provided in the figure panels and refer to the number of cells

For characterization in astrocytes and neurons, we focused on iATPSnFR^1.0^, given its better expression. We co-expressed iATPSnFR^1.0^ with mCherry in cultured U373MG astroglia and observed good cell-surface trafficking (Fig. 5a), although there was also an intracellular pool of the sensor. We confirmed that iATPSnFR^1.0^ responded to 300 µM ATP applications with fluorescence increases (Fig. 5b). Similar data were obtained by expressing iATPSnFR^1.0^ in primary cultures of rat hippocampal neurons (Fig. 5c). For U373MG astroglia, we determined the iATPSnFR^1.0^ EC_50_ to be 400 µM with a maximum d*F*/*F* of 1.1 (Fig. 5d), similar to the HEK293 cell data. In hippocampal neurons, iATPSnFR^1.0^ properly localized to membrane processes, and application of ~1 mM ATP evoked increases in fluorescence. In hippocampal neurons, the ATP EC_50_ was 630 µM and the maximum d*F*/*F* was ~1.5 (Fig. 5e). Hence, our experiences with iATPSnFR^1.0^ in solution, in HEK293 cells, in astroglia and in hippocampal neurons demonstrate that the weakening of ATP binding and the decrease in d*F*/*F* resulting from tethering the sensor to the outside of the membrane bilayer is independent of the host cell. We have observed altered sensitivity with other sensors on the membrane^26,27,31^; we speculate that it results from steric restriction from lipid head groups and membrane proteins.

We generated adeno-associated virus (AAV2/5) to express iATPSnFR^1.0^ specifically in astrocytes using the *GfaABC*_*1*_*D* promoter^38^. Following in vivo microinjections into the hippocampus, iATPSnFR^1.0^ co-localized with S100β-positive astrocytes, but not NeuN-positive neurons (Supplementary Figure 3). Bath application of ATP to multicellular preparations such as brain slices is fraught with problems caused by the rapid hydrolysis of ATP by cell surface ectoATPases. In order to minimize such effects, we puffed ATP via a glass pipette with a Picospritzer. Puffing 3 mM ATP (for 5 s) onto U373MG astroglia resulted in a ~0.7 d*F*/*F* (Fig. 5f). Similar, albeit smaller, responses were observed when we applied ATP to astrocytes expressing iATPSnFR^1.0^ in hippocampus and spinal cord slices or to D1 or D2 medium spiny neurons expressing iATPSnFR in striatum (Fig. 5g, h). Taken together, these data show that iATPSnFR can be expressed and responds reliably on the surface of cells following in vivo expression.

We next explored the use of cytosolic iATPSnFR^1.0^ to follow intracellular ATP, which ranges from 1 to 3 mM in mammalian cells^39^. We expressed cytosolic iATPSnFR^1.0^ in U373MG astroglia and detected a significant drop in intracellular fluorescence upon switching to 0 mM glucose (Supplementary Figure 4a), or by applying 10 mM 2-deoxyglucose (2DOG; Supplementary Figure 4b)—an inhibitor of glycolysis^40^. Similarly, following in vivo expression with AAVs, we measured a significant drop in intracellular ATP levels as reported by the fluorescence of iATPSnFR^1.0^ in hippocampal astrocytes in 0 mM glucose (Supplementary Figure 4c; the fluorescence of co-expressed tdTomato did not change, arguing against changes in cell shape and volume, or other non-specific artifacts, as the cause). We repeated these experiments for hippocampal astrocytes and CA1 pyramidal neurons, both at somata and along processes (Supplementary Figure 4d): oxygen-glucose deprivation (OGD) caused a substantial drop in intracellular ATP levels. We found that fluorescence decreases of astrocytes were twice that of neurons (Supplementary Figure 4d). Such responses were completely reversible for astrocytes, but only partly reversible for neurons (Supplementary Figure 4d). Furthermore, it took longer for decreases in astrocyte ATP to begin (~10 min in OGD). However, once ATP began to decrease in astrocytes, it took 8.0 ± 0.5 min (*n* = 37) to decrease by 50%, whereas in CA1 pyramidal neuron somata and dendrites it took 13.5 ± 0.8 min (*n* = 26) and 17.4 ± 0.9 min (*n* = 27), respectively (*p* < 0.0001 by ANOVA and Dunnett post hoc tests, here ± indicates plus/minus standard error of the mean). Based on the titrations in Fig. 3, we estimate that ATP levels fell by ~0.5 mM from >1 mM. Thus during glucose deprivation, ATP levels in astrocytes fall quickly, but recover completely upon restoration of glucose. In contrast, neuronal ATP levels fall slowly and fail to recover to normal levels under the identical conditions.

### mRuby-iATPSnFR^1.0^ as a ratiometric ATP sensor

**Fig. 6:**
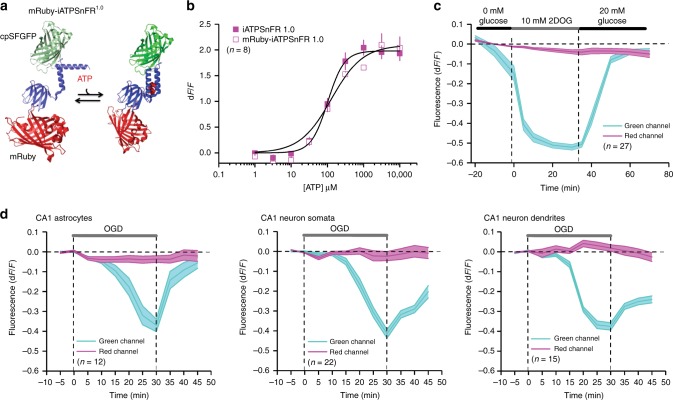
Characterization of cytosolic mRuby-iATPSnFR^1.0^. **a** Cartoon schematic of mRuby-iATPSnFR^1.0^: ATP binding is schematized to show an increase in cpSFGFP fluorescence, whereas mRuby fluorescence remains unchanged. **b** Dose–response curves for iATPSnFR^1.0^ in relation to mRuby-iATPSnFR^1.0^ in HEK293 cell lysates. **c** Changes in fluorescence of mRuby-iATPSnFR^1.0^ (green and red channels) before, during, and after inhibition of glycolysis with 2DOG in HEK293 cells. **d** Changes in fluorescence of mRuby-iATPSnFR^1.0^ (green and red channels) during oxygen-glucose deprivation in hippocampal brain slices. Data from astrocytes as well as CA1 pyramidal neuronal cell bodies and dendrites are shown. The error bars represent the s.e.m. and in some cases are smaller than the symbols used for the mean. *n* numbers are provided in the figure panels and refer to the number of cells

**Fig. 7:**
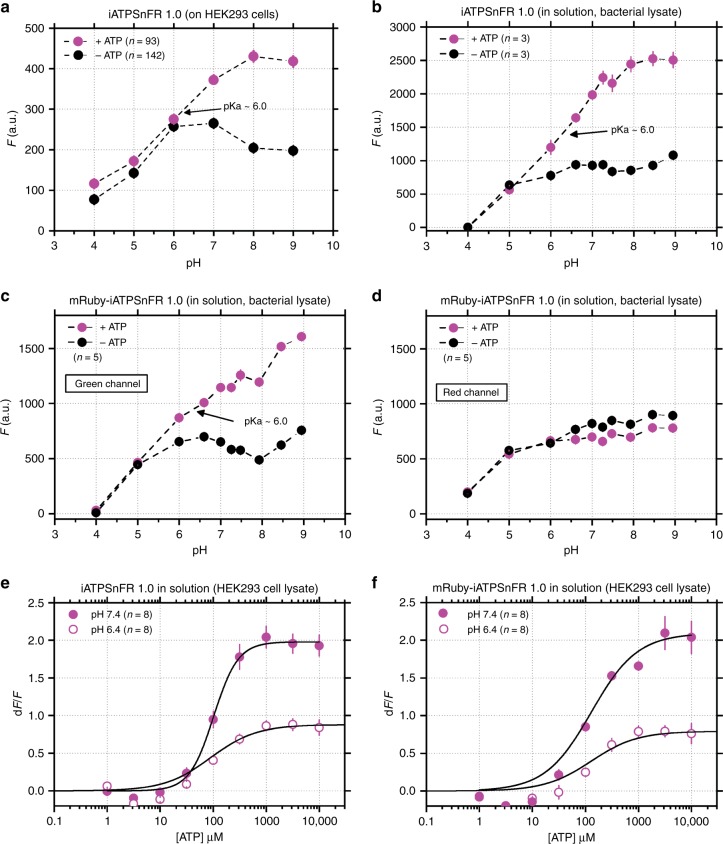
Assessments of how iATPSnFR^1.0^ was affected by pH. **a–d** Effect of pH on the unbound and ATP bound fluorescence of the indicated constructs. **e**, **f** Comparison of the effect of ATP on the fluorescence of the indicated constructs at pH 6.4 and 7.4. The error bars represent the s.e.m. and in some cases are smaller than the symbols used for the mean. *n* numbers are provided in the figure panels and refer to the number of cells in panel (**a**), and number of independent evaluations in (**b**–**f**)

We fused the red fluorescent protein mRuby^41^ to the N-terminus of iATPSnFR^1.0^ to generate a ratiometric sensor (Fig. 6a; mRuby-iATPSnFR^1.0^). The mRuby-iATPSnFR^1.0^ construct was identical to iATPSnFR^1.0^ in terms of its sensitivity and ATP-evoked d*F*/*F* (Fig. 6b). In HEK293 cells, mRuby-iATPSnFR^1.0^ displayed clear changes in green fluorescence in the presence of 2DOG, whereas the red fluorescence of mRuby did not (Fig. 6c; Supplementary Figure 5). Note that in Supplementary Figure 5, a field of view is shown, but that the analyses were performed on single cells and not by averaging across whole fields of view. Similarly, following in vivo expression with AAVs, we measured a significant drop in intracellular ATP as reported by the fluorescence of iATPSnFR^1.0^ in hippocampal astrocytes and pyramidal neuron somata and dendrites upon switching to solutions that mimic ischemia (i.e., OGD), whereas there was no change in mRuby (Fig. 6d; Supplementary Figure 6). Interestingly, ATP levels returned back to normal levels upon reapplication of physiological buffers in astrocytes, but remained low in neuronal somata and dendrites (Fig. 6d). We also performed two necessary control experiments. First, we tested whether the iATPSnFR^1.0^ itself responded to 2DOG with a decrease in fluorescence. This was not the case (Supplementary Figure 7). Second, we measured intracellular pH changes in HEK293 cells during 2DOG applications. Using confocal microscopy (Ex 473 nm, Em 525 nm), we found subtle 10% decreases in the fluorescence of the pH indicator BCECF, which suggests acidification of the intracellular compartment by ~0.2 pH units. However, this acidification took ~45 min to reach significance in the presence of 2DOG and was not reversible upon reapplication of glucose (Supplementary Figure 7b). To explore pH changes further, we also used epifluorescence dual-wavelength ratiometric imaging with BCECF-AM (Ex 505 and 440 nm, Em 535 nm), and found the 505/440 ratio decreased from 1.3 ± 0.03 to 1.2 ± 0.03 after 20 min of 2DOG, but did not recover upon reapplication of glucose (1.1 ± 0.02) for 20 min (*n* = 144). Hence, consistent with past work using 2DOG^20^ we measured variable and subtle acidification in HEK293 cells with 2DOG applications, but these changes could not account for the drop in mRuby-iATPSnFR^1.0^ fluorescence, which we associate with a drop in ATP concentration that has been previously reported^20^. For example, from calibration experiments reported in Fig. 7c (see next section), acidification from pH 7.4 to 7.2 is expected to cause a 9% decrease in fluorescence of ATP-bound mRuby-iATPSnFR^1.0^, whereas with 2DOG applications we measured a change of between 40 and 50% (Fig. 6). Thus, in accord with past studies^20^ such putative ATP changes were larger, faster and reversible in HEK293 cells (Fig. 6c). We could not use BCECF as a pH indicator for brain slice experiments because our past work shows that membrane-permeant acetoxymethyl ester (AM)-version organic dyes are poorly/unreliably taken up by adult brain slices^38,42^. Overall, mRuby-iATPSnFR^1.0^ experiments suggest that this sensor can be used as a ratiometric sensor of cell-specific intracellular ATP levels, with the caveat that parallel experiments to assess pH changes need to be performed on a case-by-case basis whenever mRuby-iATPSnFR^1.0^ is used. We next assessed pH sensitivity of the sensors directly.

### pH sensitivity of iATPSnFR^1.0^ and mRuby-iATPSnFR^1.0^

As with all GFP-based sensors^43^, the fluorescence of iATPSnFRs is expected to be sensitive to pH with a H^+^ dependence largely determined by GFP. We performed a specific set of experiments to assess this as a guide for future use of the sensors. We focused on iATPSnFR^1.0^, because this was used for most of the work reported in this study. First, we measured the fluorescence of extracellularly displayed iATPSnFR^1.0^ on the surface of HEK293 cells at a range of pH values between 4 and 9 in the absence and presence of ATP (Fig. 7a). As expected, acidification strongly decreased fluorescence of the ATP bound and unbound states. Between pH 6.5 and 9, there was a strong increase in fluorescence upon binding ATP. Second, we repeated the aforementioned experiments with iATPSnFR^1.0^ in solution (from bacterial lysates). We found the same effect with acidification for the ATP bound and unbound states (Fig. 7b). Third, we performed similar experiments for the mRuby-iATPSnFR^1.0^ sensor in solution (from bacterial lysates) while assessing fluorescence emission in the green ATP sensor channel and in the reference red mRuby channel (Fig. 7c, d). For the ATP sensors, we again found that acidification decreased fluorescence of the ATP bound and unbound states. mRuby pH effects were not ATP-dependent, indicating that mRuby-iATPSnFR^1.0^ can be used as a ratiometric sensor (Fig. 7c, d). Fourth, we performed ATP dose–response curves for iATPSnFR^1.0^ and mRuby-iATPSnFR^1.0^ in solution at pH 6.4 and 7.4 (Fig. 7e, f). These data showed that acidification decreased fluorescence responses at all concentrations of ATP that were effective in increasing fluorescence. Overall, taken together these data show that iATPSnFR^1.0^ and mRuby-iATPSnFR^1.0^ function as single wavelength and ratiometric ATP sensors, but do indeed display sensitivity to acid pH as expected by the nature of GFP^43^. Perhaps more usefully, the calibration curves shown in Fig. 7 provide a metric by which to judge the consequences of acidification in future experiments in which the sensors are used. The sensors are likely to be of limited use in environments where the pH is more acidic than ~6.2. Finally, we assessed the kinetics for ATP binding to iATPSnFR^1.0^ in solution at pH 7.4 and found that they did not follow typical pseudo-first order behavior (Supplementary Figure 8), but were instead consistent with a mechanism in which both higher and lower fluorescent states occur (potentially resulting from monomeric and dimeric states). This possibility could be usefully explored in follow up studies.

## Discussion

In the present study, we used the bright, stable cpSFGFP scaffold, rational design, and library screening to direct the selection of ATP sensors with desired characteristics. We characterized the ATP sensors to monitor cell surface and cytosolic ATP levels with excellent specificity. The current sensors may be useful to study cytosolic ATP levels and signals associated with P2X7 receptors, which respond to ATP in the hundreds of micromolar to millimolar range^44^, but they seem unsuitable to measure ATP release from astrocytes which is likely in the hundreds of nanomolar to micromolar range^45^. The sensors may also be useful for diagnostics to sense high ATP levels in tumors^46^. Strategies for further refinements are considered in the sections that follow.

We have tabulated the properties of iATPSnFRs in relation to those of existing fluorescence-based ATP sensors in Supplementary Table 1. Intracellular ATP levels are typically in the millimolar range and change during pathology. Extracellular ATP levels are usually low in physiological settings due to ectoATPases, but several decades of physiological studies and the existence of cell surface ATP receptors with sensitivity to ATP between nanomolar and millimolar indicates that extracellular ATP levels also increase during specific processes such as purinergic transmission^5,47–52^. Considering these facts, it seems unfeasible that a single sensor will be suitable to monitor intracellular and extracellular ATP levels over a concentration range spanning 5–6 orders of magnitude. Thus, ATP sensors will need to be designed for specific applications from those that are available (Supplementary Table 1) and those reported herein.

It is clear that to better detect small ATP fluctuations in living animals, the sensitivity, d*F*/*F* and kinetics of iATPSnFRs should be improved, which could be achieved using directed evolution strategies by employing the iATPSnFR^1.0^ scaffold we have reported. Important targets for such mutagenesis include the linker regions and the ATP-binding module itself. Furthermore, it may be possible to use the same scaffold to design sensors for other nucleotides and nucleosides, as with the development of NADPH sensors for example^53^. Several such strategies are underway. In these regards, progress with the generation of other sensors such those for Ca^2+^, glutamate and voltage are illuminating: the best available indicators were all generated after several years and iteratively after a useful starting point scaffold and strategy was reported. It is feasible that the development of ATP sensors will follow a similar route based on the iATPSnFR^1.0^ scaffold, or others. In parallel, however, ATP G-protein coupled receptors (GPCRs) that display a range of ATP affinities and ligand selectivities^47^ could potentially be exploited to design ATP sensors, as suggested by recent impressive strides made with the generation of acetylcholine and dopamine sensors based on their cognate GPCRs^28–30^. In evaluating these approaches, it is possible that GPCR-based sensors have the potential to form heteromers^54^ with endogenous GPCRs, whereas for sensors employing engineered scaffolds such as iATPSnFRs this possibility is less likely. Finally, GFP-based ATP sensors will likely find many uses to study the dynamics of ATP and should be considered complementary to the use of luciferase-based ATP imaging approaches, which have been shown to work for intra- and extracellular ATP in cells and in vivo^16–19,32^. For example, it seems unlikely that fluorescence-based ATP sensors can be used for whole-rodent imaging, which is possible for luciferase-based sensors^19^. Hence, the choice of sensor to employ will largely depend on the specific question being addressed and careful assessment of the contributions of pH to the signals will need to be made on a case-by-case basis.

Overall, the iATPSnFRs reported herein and the future developments they permit (to tailor sensitivity and kinetics to specific applications) portend the direct imaging of purinergic signaling^5^ throughout the body of transgenic animals. Our constructs are available (Supplementary Table 2) to allow such studies to commence in parallel with existing approaches discussed in earlier sections.

## Methods

### Cloning

The QUEEN-7µ coding sequence was purchased as a gBlock Gene Fragment from Integrated DNA Technologies with a *BglII* restriction site preceding the coding sequence of QUEEN-7µ and a *PstI* restriction site following the coding sequence. This gBlock was cloned into an in-house modified pRSET-A vector (ThermoFisher: V35120), termed pHHM.pHHM contained an N-terminal His6Gly tag utilized for purification, followed by a hemagglutinin (HA) epitope tag and a multiple cloning site before the gene of interest, as well as a myc tag at the C-terminus of the gene. This vector was used for screening, because it has the HA and myc tags present in pDisplay (ThermoFisher: V66020), which is the vector we planned to use for surface expression in mammalian cells^26,55^. Cloning from pHHM to pDisplay or into pDisplayMini, an in-house vector that lacks the N-terminal HA epitope present in pDisplay, was achieved via a restriction digest of both the iATPSnFR and pDisplay plasmids with *BglII* and *PstI*. Cloning into the pZAC-AAV-*GfaABC*_*1*_*D* was done using PCR amplification to add an *NheI* restriction site to the N-terminus of iATPSnFR and a *NotI* restriction site to the C-terminus of iATPSnFR coding sequence. Cloning was performed using routine subcloning techniques. Note that the mRuby we used was mRuby3 minus MDELYK from the C-terminus.

### Protein expression and purification

Vectors containing iATPSnFR variants were transformed into *Escherichia coli* BL21(DE3) cells (lacking pLysS). Proteins were expressed by growing the *E. coli* in liquid auto-induction media^56^ supplemented with 100 μg/ml ampicillin at 30 °C for at least 18 h. Following expression, the bacteria were harvested at 1500*g* for 20 min. The pellet was resuspended in PBS with 1 M NaCl and frozen. Protein was extracted using freeze–thaw lysis and sonication. Upon thawing, 1 µM PMSF was added to the solution before sonication. Following sonication, the crude lysate was initially clarified at 4000*g* for 20 min. The supernatant was then isolated and further clarified by centrifugation at 35,000*g* for 45 min. Proteins were purified using Ni-NTA agarose affinity chromatography (5 ml column). The iATPSnFR proteins were eluted with a 120 ml gradient from 0 to 200 mM imidazole at 2 ml/min. A blue light box was used to determine which fractions were fluorescent. The fluorescent fractions were combined and concentrated down to 2–3 ml. The protein was then dialyzed with a minimum of 1 L PBS for at least 24 h. Auto-induction media consisted of ~900 ml of ZY media (1% tryptone and 0.5% yeast extract) with 20 ml of 50× M (25 mM Na_2_HPO_4_, 25 mM KH_2_PO_4_, 50 mM NH_4_Cl and 5 mM Na_2_SO_4_), 20 ml of 50 × 5052 (0.5% glycerol, 0.05% glucose, 0.2% α-lactose), 2 ml 1 M MgSO_4_, and 200 µl of 1000× trace metals (50 mM FeCl_3_, 20 mM CaCl_2_, 10 mM MnCl_2_, 10 mM ZnSO_4_, 2 mM CoCl_2_, 2 mM CuCl_2_, 2 mM NiCl_2_, 2 mM Na_2_MoO_4_, 2 mM Na_2_SeO_3_, 2 mM H_3_BO_3_).

### Protein titrations with ATP

After protein expression, 100 µl of protein was transferred to flat-bottom black 96-well plates (Greiner). Initial fluorescence was assayed in a Safire2 plate-reading fluorimeter with a stacker (Tecan). Excitation/emission wavelengths were 485/515 nm. Gain was between 80 and 120 V and bandpass filters were 5 nm. Fluorescence was assayed again after the addition of 10 μl ATP solution containing between 315 nM and 100 mM ATP for final ATP concentrations of 31.5 nM–10 mM. After a second reading, d*F*/*F* was calculated.

For experiments performed with clarified HEK-293 lysate, 10 cm dishes were transfected with the cytoplasmic gene of interest. 24 h following transfection the cells were washed twice with PBS. A third wash of PBS was used to detach the cells. The cells were then pelleted and protein were extracted by freeze/thaw lysis. The pH of the lysate was adjusted appropriately and the lysate was concentrated to 1.5 ml using an Amicon centrifugal filter. After the lysate was concentrated, dose–response curves were generated as above.

For determining the pH dependence of fluorescence, buffered solutions of specific pH were made (Hydrion Chemvelope) and concentrated, purified protein was added to a final concentration of 0.2 µM. Fluorescence of 100 µl triplicate samples was measured as above. ATP was added (from a 200 mM stock of Mg ATP) to a final concentration of 6 mM and fluorescence was measured again.

### Stopped-flow kinetics

Equal volumes of 0.2 µM purified protein in Mg-containing buffer (10 mM HEPES, 145 mM NaCl, 2.5 mM KCl, 10 mM Glucose, 2 mM CaCl_2_, 1 mM MgCl_2_, pH 7.4) and ATP-containing buffer (various concentrations) were mixed in an SX-20 stopped-flow fluorimeter (Applied Photophysics), with data points collected every millisecond. Excitation was from a 490 nm LED. Emission was detected with a photomultiplier tube and a 510 nm long pass filter. Mixing reactions were performed in quintuplicate and averaged. Data was fit to an equation containing two exponential rises:

$$F = F_0 + \Delta F_{{\mathrm{max}}1}\ast \left( {1 - {\mathrm{exp}}\left( { - {k}_{{\mathrm{obs}}1}\ast {\mathrm{time}}} \right)} \right) + \Delta F_{{\mathrm{max}}2}\ast \left( {1 - {\mathrm{exp}}\left( { - k_{{\mathrm{obs}}2}\ast {\mathrm{time}}} \right)} \right)$$

### Mutagenesis

Linker variants and point mutations were generated by site-directed Kunkel mutagenesis^57^ with a uracil template. CJ236 cells were transformed with promising vectors and grown on plates containing chloramphenicol overnight at 37 °C. The following day a 1 ml culture of LB with only ampicillin was inoculated with a colony and grown at 37 °C with shaking until cloudy—usually about 6 h. Once the culture was cloudy, 1 µl of M13KO7 helper phage (NEB: N0315S) was added. After incubating for an additional hour, the culture was expanded to a 40 ml culture of LB with ampicillin (no chloramphenicol) in a 250 ml flask and incubated overnight at 37 °C with shaking. The following day, the bacteria were pelleted at 4000*g* for 20 min at 4 °C. The supernatant was transferred to a new 50 ml Falcon tube containing 10 ml of 20% PEG in 2.5 M NaCl. After thorough mixing, the solution was incubated on ice for 15 min. The phage was then pelleted at 4000*g* for 20 min. The supernatant was removed and the phage was resuspended in 2 ml of PBS. The 2 ml of PBS were divided into two different Eppendorf tubes and centrifuged at maximum speed for 5 min to pellet the remaining bacteria. The supernatant was transferred to two new Eppendorf tubes containing 300 µl of 20% PEG in 2.5 M NaCl. After vortexing, the solution was incubated on ice for 10 min. The phage was pelleted at maximum speed for 5 min. The supernatant was removed. Another quick spin was done to remove any residual supernatant. The phage was then resuspended in 1 ml of PBS. This was centrifuged at maximum speed for 5 min and the supernatant was transferred to a new Eppendorf tube. The uracil template was then purified using the Qiagen QIAprep Spin M13 Kit (Cat. No. 27704) using the manufacturer’s protocol. 11 µl of MP buffer was added to the 1 ml PBS/template solution. After vortexing, the mixture was incubated at room temperature for 5 min. This mixture was then applied to the QIAprep spin column and centrifuged at 4700×*g*. The flowthrough was discarded. After the entire mixture was applied, 700 µl of PB buffer was applied to the column. After 1 min of incubation, the column was centrifuged at 4700×*g* for 15 s. This was repeated before washing the column three times with 700 µl of PE buffer, and again centrifuging at 7000 rpm for 15 s. After ensuring the column was dry, 25 µl of EB was applied to the column which was then centrifuged at 4700×*g*. This was done three times for a total of 75 µl of EB. DNA quality was checked via using agarose gel electrophoresis. High-quality preparations run as a single band about 1/3 the size of the plasmid.

Degenerate primers were designed to mutate two amino acids at a time. This was done by flanking the degenerate/mutated sequence of DNA with about 15 bases of unmutated DNA on either side. A variant of QUEEN, with cpSFGFP replacing cpEGFP was cloned by PCR. Multiple rounds of mutagenesis were performed to screen variants with mutations at the junction of PS3 and cpSFGFP (Linker 1) and cpSFGFP and PS3 (Linker 2). In the following primers, X indicates the codon that was randomized to encode all possible amino acids. A Linker 2 primer encoding GHKLEYNXXDIDFKRA, resulted in a variant with the Linker 2 sequence GHKLEYNLHDIDFKRA. This was re-randomized with a number of primers (many of which did not yield improved sensors), including a primer encoding GHKLEYNFXLHXIDFKRA, from which the final Linker 2 sequence GHKLEYNFGLHDIDFKRA was identified. Upon that template, Linker 1 was optimized with a primer encoding AERRLQSQXXSHNVYIT. From a screen of a few hundred variants, the winner (and ultimate iATPSnFR^1.0^) sequence AERRLQSQVLSHNVYIT was identified. The primers were phosphorylated using T4 kinase. The reaction was performed in an Eppendorf tube containing 18 µl H_2_O, 3 µl T4 Kinase buffer, 1 µl 10 µM ATP, 1 µl T4 Kinase (NEB M0201L), and 7 µl 100 µM of the oligo template. The reaction was then incubated for 30 min at 37 °C. To determine the optimal template to primer ratio, four annealing reactions were initially set up using four different primer dilutions of the kinased primer; 1:1, 1:5, 1:10, and 1:100. 1 µl of diluted primer, 1 µl of template and 1 µl of 10× T4 DNA ligase buffer, and 7 µl of nuclease-free water was combined in a PCR tube. The reaction was heated to 95 °C for 2 min using a PCR machine. The reaction was then slowly brought back to 25 °C using 5 °C steps for 30 s each. To polymerize the DNA, 9.5 µl water, 1.5 µl 10× T4 DNA ligase buffer, 1 µl 25 mM dNTPs, 1 µl 10 mM ATP, 1 µl T7 polymerase unmodified (NEB: M0274L), 1 µl T4 ligase (NEB: M0202M), and the entire 10 µl annealing reaction was added to a PCR tube and was allowed to incubate at room temperature for an hour. This reaction was transformed directly into *E. coli* BL21(DE3) cells (lacking pLysS) and plated on 24 cm × 24 cm LB-Agar plates with 100 μg/ml ampicillin. After determining the optimal concentration of kinased oligos, that dilution can be used for subsequent reactions with that particular uracil template.

### High-throughput screening

Individual colonies were selected using a QPix2^xt^ colony-picking robot (Genetix). Colonies were deposited into deep 96-well plates filled with 800 µl auto-induction media with 100 μg/ml ampicillin. Cultures were grown with vigorous shaking overnight at 30 °C. The bacteria were harvested by centrifugation (3000*g*) and washed three times with 900 µl PBS before freezing. Protein was extracted by freeze–thaw lysis with 900 µl phosphate-buffered saline. Rapid shaking was used to homogenize the solution. Crude lysate was clarified by centrifugation at 4000*g* for 30 min. 100 µl of clarified lysate was transferred to flat-bottom black 96-well plates (Greiner) using a liquid-handling robot. Initial fluorescence was assayed in a Safire2 plate-reading fluorimeter with stacker (Tecan). Excitation/emission wavelengths were 485/515 nm. Gain was between 80 and 120 V and bandpass filters were 5 nm. Fluorescence was assayed again after the addition of 10 μl 100 mM ATP (final ATP concentration 10 mM). After calculating d*F*/*F*, new uracil templates were made for promising candidates and were subject to subsequent rounds of mutagenesis. The most promising sensors were sequenced and cloned into either pDisplay or pDisplayMini for further screening in mammalian cells.

### Transfection of HEK293 and U373 cells

HEK293 (ATCC, CRL-1573) and U373MG cells (Sigma-Aldrich, 08061901) were maintained in DMEM/F-12, supplemented with GlutaMAX (Gibco: 10565042), 10% FBS (Gibco 10082147), and 5 ml penicillin–streptomycin (10,000 U/ml) (Gibco: 15140-122) at 37 °C with 5% CO_2_. Cells were trypsinized once they reached confluence and were diluted at a ratio of 1:10. HEK-293 and U373MG cells were transfected with either Lipofectamine 2000 (ThermoFisher 11668027) or Effectene (Qiagen ID: 301425) following the manufacturer’s guidelines. The following is the procedure for the transfection of a single well of a 6-well dish. 100 µl of OptiMEM (Gibco: 31985062) was placed in two tubes. 2.5 µg of DNA was added to one tube and 6.25 µl of Lipofectamine 2000 was added to the other. The content of the two tubes was combined, thoroughly mixed, and incubated for 20 min at room temperature before adding the mixture to cells that were 70–90% confluent with fresh media. The media was changed 6–8 h later. 0.4 µg DNA was added to a tube, followed by 8 µl of enhancer. 100 µl of pipetted Buffer EC was used to mix the contents by pipetting. The mixture was then incubated for 2 min before thoroughly mixing 8 µl of Effectene into the mixture. The solution was then incubated for 10 min. 600 µl of fresh media was added before adding the mixture to cells that were 70–90% confluent with fresh media. The media was changed the following day.

### Lysis of HEK293 cells

HEK293 cells were washed with cold PBS three times prior to lysing cells. Cells were lysed on ice using RIPA buffer: 150 mM NaCl, 20 mM Tris, 1 mM EDTA, 1% Triton, 0.1% SDS, and 0.5% sodium deoxycholate at pH 8 supplemented with protease inhibitors (ThermoFisher). Following the 10 min incubation on ice, the mixture was centrifuged at max speed for 10 min. The supernatant was taken and diluted with an equal volume of 2× Laemmli buffer for western blot analysis.

### Western blotting

Precast gels were purchased from BioRad and were run for 110 min at 100 V. 1× running buffer was composed of 3.0 g Tris base, 14.4 g glycine and 1.0 g SDS in 1 l water. Gels were transferred to nitrocellulose for 1 h at 100 V in 1× transfer buffer. 10× transfer buffer was composed of 30.0 g Tris and 144.0 g glycine in 1 l water. 1× Transfer buffer is composed of 10% 10× running buffer, 20% methanol, and 70% water. Following transfer, blots were blocked with 5% bovine serum albumin (BSA) (Sigma: A9647-100G) in TBS with 0.05% Tween-20 (TBST) (Sigma: P2287-100ML) for 1 h. Antibodies were made with 5% BSA in TBST. Primary antibodies: GAPDH 1:5000 mouse (Thermofisher MA5-15738). GFP 1:1000 rabbit (Invitrogen A11122). Secondary antibodies: IRDye 680RD anti-mouse 1:20,000 (Licor 926-68170). IRDye 800CW anti-rabbit 1:20,000 (Licor 925-68024). Primary antibodies were incubated overnight at 4 °C. Following three washes with TBST of at least 10 min each, secondary antibodies were incubated for 1 h at room temperature. Three washes with TBST of at least 10 min each were sufficient to remove the secondary antibodies. Membranes were imaged using a Licor Odyssey infrared imager.

### AAV2/5 microinjections in vivo

All animal experiments were conducted in accordance with the National Institute of Health Guide for the Care and Use of Laboratory Animals and were approved by the Chancellor’s Animal Research Committee at the University of California, Los Angeles. Wild-type C57BL/6NTac mice were generated from in house breeding colonies or purchased from Taconic Biosciences. All mice were housed with food and water available ad libitum in a 12 h light/dark environment. All animals were healthy with no obvious behavioral phenotype, were not involved in previous studies, and were sacrificed during the light cycle. Animals were initially deeply anesthetized in a chamber with 5% isofluorane. The head of the animal was then shaved to remove the fur around the surgical site. The animal had its head fixed in a stereotaxic setup using ear bars. A nose cone was placed over the nose for continuous administration of isofluorane throughout the procedure. There was continuous monitoring of the animal’s breathing during the procedure and anesthesia was adjusted accordingly (0.5–3%). The animal was administered buprenorphine (0.1 mg/kg Buprenex) and the surgical site was cleaned three times with 10% povidone iodine and 70% ethanol prior to the surgery. A small incision was made to expose the skull. A ~2 mm hole was drilled into the skull with a high-speed drill at 2 mm posterior to bregma and 1.5 mm left of bregma for hippocampus CA1, and at 0.8 mm anterior to bregma and 2 mm left of bregma and for dorsolateral striatum. After removing a bit of the skull, saline was applied to the surgical site to remove any debris. The CA1 region of the hippocampus was injected with 1 µl of virus (titer 1.5E13) over the course of 5 min. The stereotaxic coordinates used are 2 mm posterior from bregma, 1.5 mm left of the midline, and 1.6 mm from the pial surface for hippocampus CA1, and 0.8 mm anterior to bregma, 2 mm left of the midline, and 2.4 mm from the pial surface for dorsolateral striatum. Once the injection was complete, the needle remained in place for 10 min to allow the virus to diffuse into the tissue. The needle was gradually removed over the course of 2 min. The surgical site was sutured. Animals recovered in a cage placed on a heating pad. Animals were given 0.1 mg/kg Buprenex the following day for pain. Animals were monitored after recovery to make sure they were healthy, and they received trimethoprim sulfamethoxazole in their food for a week following the operation. Targeting of MSNs was achieved using a FLEX-dependent iATPSnFR^1.0^ AAV with D1 and D2 selective Cre mouse lines^58^.

### Imaging

HEK293 cells were transiently transfected with a vector encoding the IgK leader sequence followed by iATPSnFR, GGTGGS linker, a myc tag and the transmembrane domain of the PDGF receptor. After 24 h of incubation at 37 °C with 5% CO_2_ in 6-well plates, cells were trypsinized and plated onto coverslips that were pre-coated with poly-lysine. After another 24 h incubation at 37 °C with 5% CO_2_, the coverslips were mounted to a perfusion chamber. Images were acquired with an Olympus IXS 71 using an UplansApo 40 × 0.90 NA oil immersion lens or an Olympus BX51WI confocal microscope with a LUMPlanFL/IR 40 × 0.80 NA water immersion lens. Once mounted in the perfusion chamber, a constant flow of fresh HEK293 buffer was perfused over the cells before and after the application of ligands. ATP solutions were made up fresh daily. The steady-state fluorescence level was calculated as an average of the images acquired in the first 30 s. Fast-solution changes were achieved using SF-77B perfusion fast step controller from Warner Instruments. A 3-barreled glass square (Warner cat 64-0119) was placed as close to the cell as possible for imaging purposes. Steps of 800 µm were used to move the barrels onto and off the cells. On and off rates were determined using pClamp 10.6. HEK buffer: 140 mM NaCl, 1 mM MgCl_2_, 2 mM CaCl_2_, 10 mM HEPES, and 10 mM glucose adjusted to pH 7.4 with 5 mM NaOH. Intracellular pH assessments were made using BCECF-AM following the manufacturer’s instructions (Invitrogen). For confocal microscopy, we used 473 nm excitation and 525 nm emission. For epifluorescence microscopy, we used ratiometric imaging with alternating 440 (500 ms), 505 nm (50 ms) excitation, and 535 nm emission imaging. A monochromator (Till Photonics Polychrome IV or V) and a CCD were used for these experiments (Till Imago from Till Photonics) with imaging being controlled by TillVision software.

Animals were deeply anesthetized with pentobarbital (i.p.) prior to transcardial perfusion. Once no reflexes were observed, the chest cavity was opened. The left ventricle was impaled with a needle connected to a perfusion pump and the right was punctured to permit transcardial perfusion. The animals were perfused with at least 30 ml of ice-cold PBS to completely flush the blood, followed by at least 30 ml of 10% buffered formalin to fix the tissues. Tissues were gently dissected out and fixed overnight in 10% buffered formalin at 4 °C. Tissues were then cryoprotected with 30% sucrose in PBS before being frozen in O.C.T. compound (Fisher Scientific: 23-730-571). 40 µm sections were harvested using a cryostat microtome (Leica). Sections were harvested into PBS and blocked with 10% normal goat serum and 0.5% Triton-X 100 in PBS for 1 h at room temperature prior to staining. Primary antibody was applied overnight at 4 °C in PBS with 10% normal goat serum and 0.5% Triton-X 100. Sections were washed three times with PBS following primary application. The secondary antibody was incubated at room temperature for 1 h followed by three washes with PBS before mounting on to slides. Sections were allowed to completely dry on the slide before applying Fluoromount-G (SouthernBiotech: 0100-01). Primary antibodies: S100β rabbit 1:1000 (Abcam ab41548). NeuN rabbit 1:1000 (Cell Signaling D3S3I). GFP chicken 1:1000 (Abcam ab13970). Secondary antibodies: goat anti-chicken IgG Alexa 488 1:2000 (Invitrogen A11039). Goat anti-rabbit Alexa 546 1:2000 (Invitrogen A11010). A 488 nm argon laser adjusted to 5–10% maximum output intensity was used for imaging Alexa 488 dyes. A 543 HeNe laser adjusted to 20–30% maximum output intensity was used for imaging Alexa 546 dyes.

A Model P-97 pipette puller from Sutter Instrument Co. was used to pull capillaries purchased from VWR (1B100-4). Patch pipettes were filled with 3 mM ATP and Alexa Fluor 568 hydrazide (1:200) (ThermoFisher Scientific: A10441). Micromanipulators were used to place the tip of the pipette 10–20 μm above the cell being imaged. A constant flow of fresh buffer perfused the imaging chamber at all times. After 30 s of imaging, a 5 s puff of 3 mM ATP was administered via the PicoSpritzer III from Intracel.

Animals were deeply anesthetized with isofluorane prior to decapitation. The brain was removed as gently and as quickly as possible, and then cooled as quickly as possible. The brain was mounted with superglue to a cutting platform and was bathed in ice-cold hippocampal slicing buffer while 300 µm sections were harvested using a vibratome. For spinal cord slices, a laminectomy was performed. The dura mater and all roots were removed and the spinal cord was subsequently mounted using superglue on an agarose block for transversal sectioning (300 µm). These sections were then placed in 32 °C hippocampal imaging buffer. All solutions were bubbled with 95% O_2_ and 5% CO_2_ for at least 30 min prior to slicing. Following sectioning, the slices were placed in a perfusion chamber for imaging with a constant flow of 2–3 ml/min of hippocampal imaging buffer. Images were taken using an Olympus BX51WI confocal microscope with a LUMPlanFL/IR 40 × 0.80 NA water immersion lens. A 488 nm argon laser adjusted to 10–15% maximum output intensity for imaging GFP. Hippocampal slicing buffer: 87 mM NaCl, 25 mM NaHCO_3_, 25 mM glucose, 2.5 mM KCl, 1.25 mM NaH_2_PO_4_, 75 mM sucrose, 7 mM MgCl_2_, and 0.5 mM CaCl_2_. Hippocampal imaging buffer: 126 mM NaCl, 25 mM NaHCO_3_, 10 mM glucose, 2.5 mM KCl, 1.24 mM NaH_2_PO_4_, 1.30 mM MgCl_2_, and 2.4 mM CaCl_2_.

### Data analyses and statistics

Data from every experiment represents a minimum of *n* mice (for the experiments where mice were used). In all the imaging experiments, *n* was >3 independent evaluations (mice or transfections). Sample sizes were not calculated a priori. In the figure panels and legends, *n* is defined as the number of replicates (cells, dendrites, regions of interest or evaluations in the case of biochemical evaluations) and generally refers to the numbers of cells unless otherwise stated. For AAV injections, mice were randomly assigned to each experimental group. No experimental data points were excluded. Statistical tests were run in Origin 9. Summary data are presented as mean ± s.e.m. Note that in some of the graphs, the bars representing the s.e.m. are smaller than the symbols used to represent the mean. For each set of data to be compared, we determined within Origin or GraphPad InStat whether the data were normally distributed or not using the Shapiro–Wilk test. If they were normally distributed, we used parametric tests. If the data were not normally distributed, we used non-parametric tests. Paired and unpaired Student’s two-tailed *t* tests (as appropriate) and two-tailed Mann–Whitney tests were used for most statistical analyses with significance declared at *p* < 0.05. When a statistical test was employed that was not a Student’s *t* test or a Mann–Whitney test for a specific case, then it is stated as such in the text. Throughout the manuscript, the results of statistical tests (*p* values and *n* numbers) are reported in full on the figure panels to save space in the main body of the manuscript. Precise *p* values are used, but when the *p* value was less than 0.0001, it is stated at *p* < 0.0001 because the program we used does not provide precise numbers for such low values. *n* numbers are reported in the figure panels and represent discreet samples. However, where appropriate, key statistics are also reported in the text. Images were analyzed using ImageJ. The GECIquant program^59^ plugin was used to subtract the background from images as well as to select regions of interest. Mean gray value measurements were imported into Excel for d*F*/*F* analysis. The final results were graphed in OriginPro 2016 and the figures assembled in CorelDraw 2017 to generate TIFF format figure files.

### Reporting summary

Further information on experimental design is available in the Nature Research Reporting Summary linked to this article.

## Supplementary information

Supplementary Information

Description of Additional Supplementary Files

Supplementary Movie 1

Supplementary Movie 2

Reporting Summary

## Data Availability

All the data are available upon request from the authors. All the newly generated plasmids have been deposited at Addgene with accession IDs that are listed in Supplementary Table 2.
