# Supplementary material for: A genetically encoded single-wavelength sensor for imaging cytosolic and cell surface ATP
Source: Nat Commun. 2019 Feb 12;10:711. doi: 10.1038/s41467-019-08441-5 (PMC6372613; doi:10.1038/s41467-019-08441-5)
Supplement: Supplementary file 1 — Supplementary Information [file 41467_2019_8441_MOESM1_ESM.pdf]

A genetically encoded single-wavelength sensor for imaging cytosolic and cell surface ATP

Lobas et al.,

Ig-kappa leader sequence | GFP | PDGFR 513-561
epsilon subunit | GGTGGS linker | **Mutations**
Linkers 1 and 2 | Myc epitope | **ATP-binding pocket**

```
                  M  E  T  D  T  L  L  L  W  V  L  L  L  W  V  P  G  S  T  G
iATPSnFR1.0 1     ATGGAGACAGACACACTCCTGCTATGGGTACTGCTGCTCTGGGTTCCAGGTTCCACTGGT  60
                  ||||||||||||||||||||||||||||||||||||||||||||||||||||||||||||
iATPSnFR1.1 1     ATGGAGACAGACACACTCCTGCTATGGGTACTGCTGCTCTGGGTTCCAGGTTCCACTGGT  60

                  D  R  S  M  K  T  I  H  V  S  V  V  T  P  D  G  P  V  Y  E
iATPSnFR1.0 61    GACAGATCTATGAAGACTATTCACGTGAGTGTCGTAACTCCCGACGGGCCTGTATATGAG  120
                  ||||||||||||||||||||||||||||||||||||||||||||||||||||||||||||
iATPSnFR1.1 61    GACAGATCTATGAAGACTATTCACGTGAGTGTCGTAACTCCCGACGGGCCTGTATATGAG  120

                  D  D  V  E  M  V  S  V  K  A  K  S  G  E  L  G  I  L  P  G
iATPSnFR1.0 121   GATGACGTTGAAATGGTGAGCGTCAAAGCAAAAAGTGGCGAGCTCGGTATTCTCCCAGGC  180
                  ||||||||||||||||||||||||||||||||||||||||||||||||||||||||||||
iATPSnFR1.1 121   GATGACGTTGAAATGGTGAGCGTCAAAGCAAAAAGTGGCGAGCTCGGTATTCTCCCAGGC  180

                  H  I  P  L  K  A  P  L  E  I  S  A  A  R  L  K  K  G  G  K
iATPSnFR1.0 181   CACATTCCCTTGAAAGCTCCCCTAGAGATCAGTGCCGCACGCCTGAAGAAAGGGGGCAAA  240
                  ||||||||||||||||||||||||||||||||||||||||||||||||||||||||||||
iATPSnFR1.1 181   CACATTCCCTTGAAAGCTCCCCTAGAGATCAGTGCCGCACGCCTGAAGAAAGGGGGCAAA  240

                  T  Q  Y  I  A  V  S  G  G  N  L  E  V  R  P  D  K  V  T  I
iATPSnFR1.0 241   ACACAGTATATCGCTGTGTCAGGCGGCAACCTCGAAGTGCGGCCTGACAAGGTGACCATC  300
                  ||||||||||||||||||||||||||||||||||||||||||||||||||||||||||||
iATPSnFR1.1 241   ACACAGTATATCGCTGTGTCAGGCGGCAACCTCGAAGTGCGGCCTGACAAGGTGACCATC  300

                  Y  A  Q  A  A  E  R  A  E  D  I  D  V  L  R  A  K  A  A  K
iATPSnFR1.0 301   TATGCCCAGGCAGCCGAGAGGGCTGAGGATATCGATGTCCTGCGCGCCAAGGCCGCCAAG  360
                  |||||||||||||||||||||||||||||||||||||||||||||||||||   ||||||
iATPSnFR1.1 301   TATGCCCAGGCAGCCGAGAGGGCTGAGGATATCGATGTCCTGCGCGCCAAGAAAGCCAAG  360
                                                                     K
                  E  R  A  E  R  R  L  Q  S  Q  V  L  S  H  N  V  Y  I  T  A
iATPSnFR1.0 361   GAGAGAGCCGAGCGCCGACTCCAATCACAGGTCCTGAGCCACAACGTCTATATCACCGCC  420
                  ||||||||||||||||||||||||||||||||||||||||||||||||||||||||||||
iATPSnFR1.1 361   GAGAGAGCCGAGCGCCGACTCCAATCACAGGTCCTGAGCCACAACGTCTATATCACCGCC  420

                  D  K  Q  K  N  G  I  K  A  N  F  K  I  R  H  N  V  E  D  G
iATPSnFR1.0 421   GACAAGCAGAAGAACGGCATCAAGGCGAACTTCAAGATCCGCCACAACGTGGAGGACGGC  480
                  ||||||||||||||||||||||||||||||||||||||||||||||||||||||||||||
iATPSnFR1.1 421   GACAAGCAGAAGAACGGCATCAAGGCGAACTTCAAGATCCGCCACAACGTGGAGGACGGC  480

                  S  M  Q  L  A  D  H  Y  Q  Q  N  T  P  I  G  D  G  P  V  L
iATPSnFR1.0 481   AGCATGCAGCTCGCCGACCACTACCAGCAGAACACCCCCATCGGCGACGGCCCCGTGCTG  540
                  ||||||||||||||||||||||||||||||||||||||||||||||||||||||||||||
iATPSnFR1.1 481   AGCATGCAGCTCGCCGACCACTACCAGCAGAACACCCCCATCGGCGACGGCCCCGTGCTG  540

                  L  P  D  N  H  Y  L  S  T  Q  S  V  L  S  K  D  P  N  E  K
iATPSnFR1.0 541   CTGCCCGACAACCACTACCTGAGCACCCAGTCCGTGCTGAGCAAAGACCCTAACGAGAAG  600
                  ||||||||||||||||||||||||||||||||||||||||||||||||||||||||||||
iATPSnFR1.1 541   CTGCCCGACAACCACTACCTGAGCACCCAGTCCGTGCTGAGCAAAGACCCTAACGAGAAG  600


                  R  D  H  M  V  L  L  E  F  V  T  A  A  G  I  T  L  G  M  D
iATPSnFR1.0 601   CGCGATCACATGGTCCTGCTGGAGTTCGTGACCGCCGCCGGGATCACTCTCGGCATGGAC  660
                  ||||||||||||||||||||||||||||||||||||||||||||||||||||||||||||
iATPSnFR1.1 601   CGCGATCACATGGTCCTGCTGGAGTTCGTGACCGCCGCCGGGATCACTCTCGGCATGGAC  660
```

```
                  E  L  Y  K  G  G  T  G  G  S  M  S  K  G  E  E  L  F  T  G
iATPSnFR1.0 661   GAGCTGTACAAGGGCGGTACCGGAGGGAGCATGAGCAAGGGCGAGGAGCTGTTCACCGGG   720
                  ||||||||||||||||||||||||||||||||||||||||||||||||||||||||||||
iATPSnFR1.1 661   GAGCTGTACAAGGGCGGTACCGGAGGGAGCATGAGCAAGGGCGAGGAGCTGTTCACCGGG   720

                  V  V  P  I  L  V  E  L  D  G  D  V  N  G  H  K  F  S  V  R
iATPSnFR1.0 721   GTGGTGCCCATCCTGGTCGAGCTGGACGGCGACGTAAACGGCCACAAGTTCAGCGTGCGC   780
                  ||||||||||||||||||||||||||||||||||||||||||||||||||||||||||||
iATPSnFR1.1 721   GTGGTGCCCATCCTGGTCGAGCTGGACGGCGACGTAAACGGCCACAAGTTCAGCGTGCGC   780

                  G  E  G  E  G  D  A  T  N  G  K  L  T  L  K  F  I  C  T  T
iATPSnFR1.0 781   GGCGAGGGCGAGGGCGATGCCACCAACGGCAAGCTGACCCTGAAGTTCATCTGCACCACC   840
                  ||||||||||||||||||||||||||||||||||||||||||||||||||||||||||||
iATPSnFR1.1 781   GGCGAGGGCGAGGGCGATGCCACCAACGGCAAGCTGACCCTGAAGTTCATCTGCACCACC   840

                  G  K  L  P  V  P  W  P  T  L  V  T  T  L  T  Y  G  V  Q  C
iATPSnFR1.0 841   GGCAAGCTGCCCGTGCCCTGGCCCACCCTCGTGACCACCCTGACCTACGGCGTGCAGTGC   900
                  ||||||||||||||||||||||||||||||||||||||||||||||||||||||||||||
iATPSnFR1.1 841   GGCAAGCTGCCCGTGCCCTGGCCCACCCTCGTGACCACCCTGACCTACGGCGTGCAGTGC   900

                  F  S  R  Y  P  D  H  M  K  Q  H  D  F  F  K  S  A  M  P  E
iATPSnFR1.0 901   TTCAGCCGCTACCCCGACCACATGAAGCAGCACGACTTCTTCAAGTCCGCCATGCCCGAA   960
                  ||||||||||||||||||||||||||||||||||||||||||||||||||||||||||||
iATPSnFR1.1 901   TTCAGCCGCTACCCCGACCACATGAAGCAGCACGACTTCTTCAAGTCCGCCATGCCCGAA   960

                  G  Y  V  Q  E  R  T  I  S  F  K  D  D  G  T  Y  K  T  R  A
iATPSnFR1.0 961   GGCTACGTCCAGGAGCGCACCATCAGCTTCAAGGACGACGGCACCTACAAGACCCGCGCC   1020
                  ||||||||||||||||||||||||||||||||||||||||||||||||||||||||||||
iATPSnFR1.1 961   GGCTACGTCCAGGAGCGCACCATCAGCTTCAAGGACGACGGCACCTACAAGACCCGCGCC   1020

                  E  V  K  F  E  G  D  T  L  V  N  R  I  E  L  K  G  I  D  F
iATPSnFR1.0 1021  GAGGTGAAGTTCGAGGGCGACACCCTGGTGAACCGCATCGAGCTGAAGGGCATCGACTTC   1080
                  ||||||||||||||||||||||||||||||||||||||||||||||||||||||||||||
iATPSnFR1.1 1021  GAGGTGAAGTTCGAGGGCGACACCCTGGTGAACCGCATCGAGCTGAAGGGCATCGACTTC   1080

                  K  E  D  G  N  I  L  G  H  K  L  E  Y  N  F  G  L  H  D  I
iATPSnFR1.0 1081  AAGGAGGACGGCAACATCCTGGGGCACAAGCTGGAGTACAACTTTGGGTTGCACGACATC   1140
                  ||||||||||||||||||||||||||||||||||||||||||||||||||||||||||||
iATPSnFR1.1 1081  AAGGAGGACGGCAACATCCTGGGGCACAAGCTGGAGTACAACTTTGGGTTGCACGACATC   1140

                  D  F  K  R  A  E  L  A  L  K  R  A  M  N  R  L  S  V  A  E
iATPSnFR1.0 1141  GATTTTAAGCGCGCCGAGCTCGCCCTTAAGCGCGCAATGAATAGGCTCTCAGTTGCCGAA   1200
                  |||||||||||||||||||||   ||||||||||||||||||||||||||||||||||||
iATPSnFR1.1 1141  GATTTTAAGCGCGCCGAGCTCAGCCTTAAGCGCGCAATGAATAGGCTCTCAGTTGCCGAA   1200
                                       S
                  M  K  G  G  T  G  G  S  L  Q  V  D  E  Q  K  L  I  S  E  E
iATPSnFR1.0 1201  ATGAAGGGAGGGACCGGCGGTAGCCTGCAGGTCGACGAACAAAAACTCATCTCAGAAGAG   1260
                  ||||||||||||||||||||||||||||||||||||||||||||||||||||||||||||
iATPSnFR1.1 1201  ATGAAGGGAGGGACCGGCGGTAGCCTGCAGGTCGACGAACAAAAACTCATCTCAGAAGAG   1260

                  D  L  N  A  V  G  Q  D  T  Q  E  V  I  V  V  P  H  S  L  P
iATPSnFR1.0 1261  GATCTGAATGCTGTGGGCCAGGACACGCAGGAGGTCATCGTGGTGCCACACTCCTTGCCC   1320
                  ||||||||||||||||||||||||||||||||||||||||||||||||||||||||||||
iATPSnFR1.1 1261  GATCTGAATGCTGTGGGCCAGGACACGCAGGAGGTCATCGTGGTGCCACACTCCTTGCCC   1320


                  F  K  V  V  V  I  S  A  I  L  A  L  V  V  L  T  I  I  S  L
iATPSnFR1.0 1321  TTTAAGGTGGTGGTGATCTCAGCCATCCTGGCCCTGGTGGTGCTCACCATCATCTCCCTT   1380
                  ||||||||||||||||||||||||||||||||||||||||||||||||||||||||||||
iATPSnFR1.1 1321  TTTAAGGTGGTGGTGATCTCAGCCATCCTGGCCCTGGTGGTGCTCACCATCATCTCCCTT   1380
```

```
                   I  I  L  I  M  L  W  Q  K  K  P  A  *
iATPSnFR1.0 1381  ATCATCCTCATCATGCTTTGGCAGAAGAAGCCACGTTAG  1419
                  |||||||||||||||||||||||||||||||||||||||
iATPSnFR1.1 1381  ATCATCCTCATCATGCTTTGGCAGAAGAAGCCACGTTAG  1419
```

Supplementary Figure 1 Sequence alignment of the iATPSnFR$^{1.0}$ and iATPSnFR$^{1.1}$ cDNAs. The sequences were aligned with Vector NTI and have been deposited at Addgene with the plasmids (see Supplementary Table 2 for plasmid IDs).

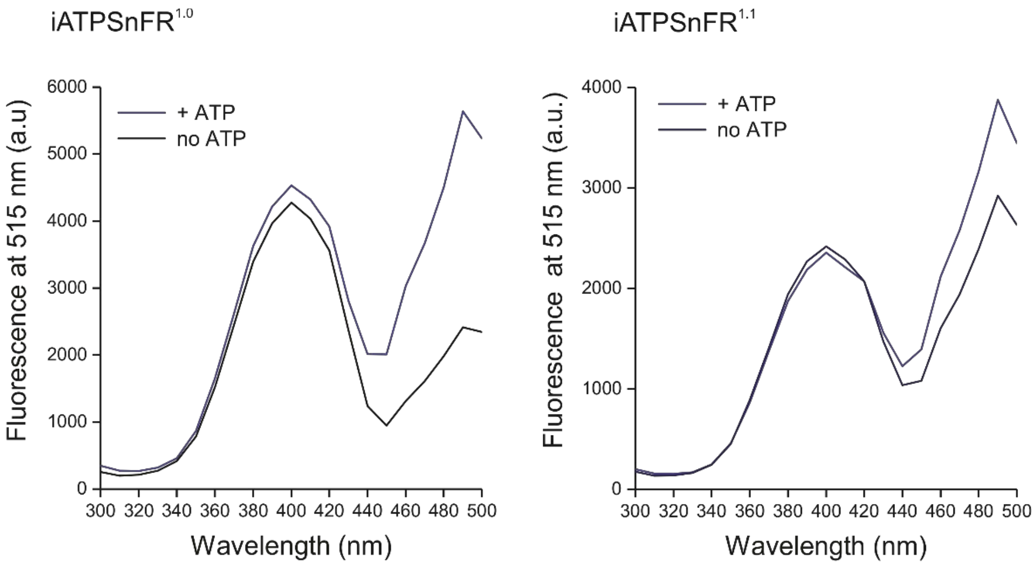

Supplementary Figure 2 Excitation spectra for iATPSnFR$^{1.0}$ and iATPSnFR$^{1.1}$ shown over an extended range in relation to those reported in Figure 1 of the main manuscript (the traces are the average from 48 replicates each in a 96 well plate).

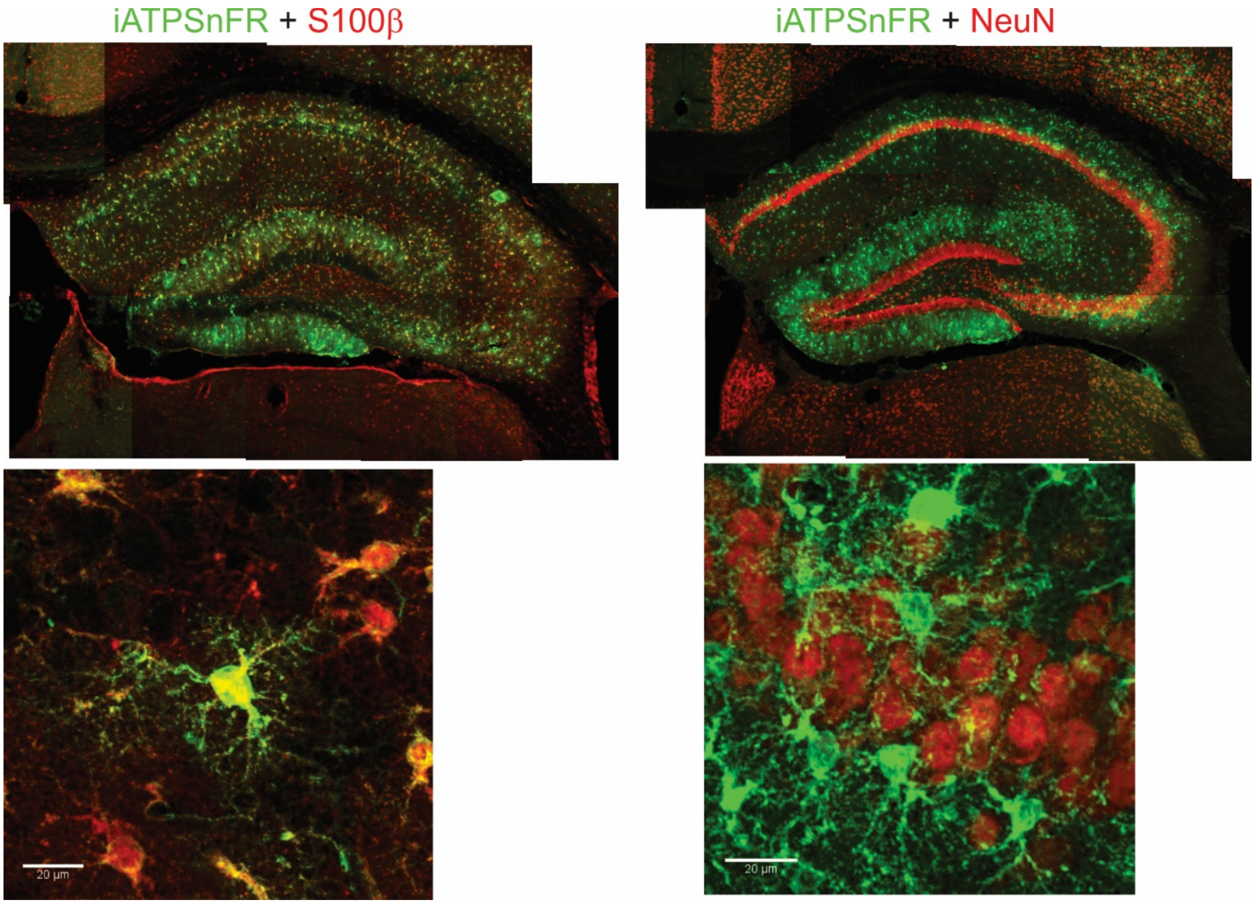

Supplementary Figure 3 iATPSnFR$^{1.0}$ was expressed in S100β-positive astrocytes, but not neurons following *in vivo* expression with AAV2/5 and the *GfaABC$_1$D* promoter. The representative images are from 4 similar mice. Note also that iATPSnFR$^{1.0}$ was found throughout the astrocyte's processes.

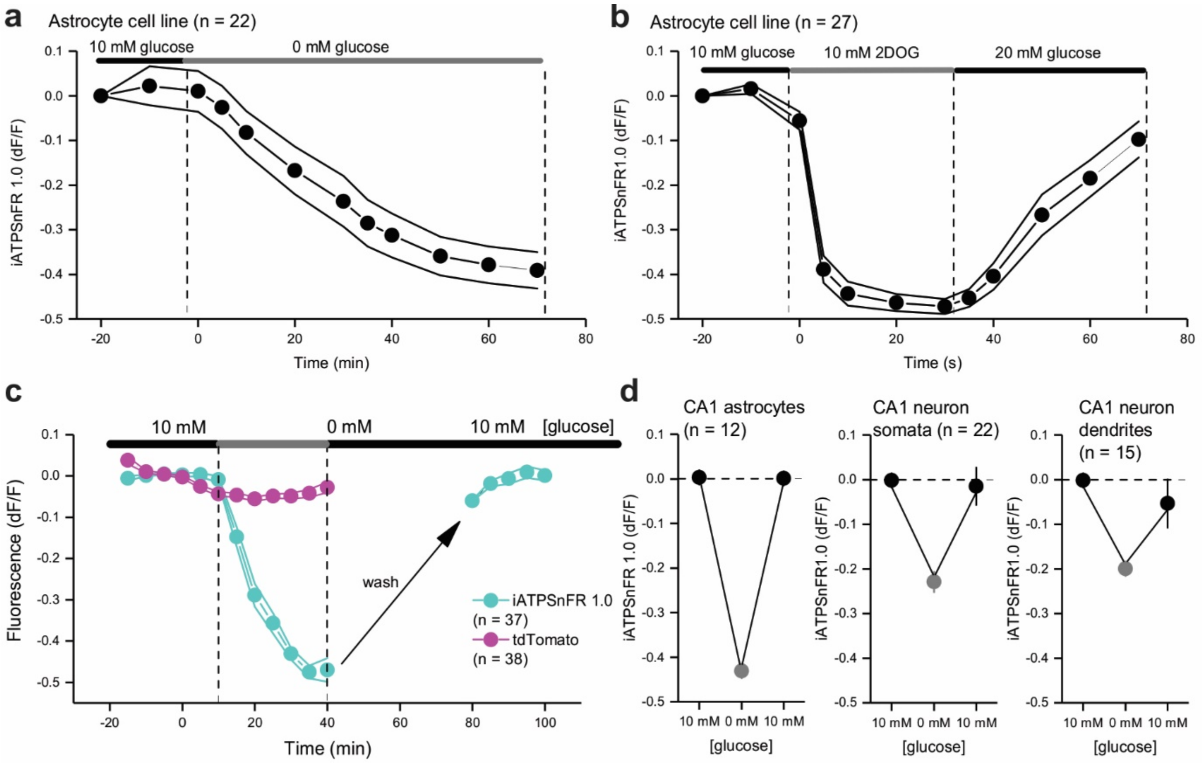

Supplementary Figure 4 Responses of cytosolic iATPSnFR$^{1.0}$. **(a)** Drop in fluorescence in U373MG astroglia during 0 mM glucose applications. **(b)** Drop in fluorescence in U373MG astroglia during 2DOG to reduce glycolysis. **(c)** As in (a), but for expression of iATPSnFR$^{1.0}$ in hippocampal astrocytes with AAV. **(d)** Summary from experiments such as those in (c) for hippocampal astrocytes, CA1 pyramidal neurons, and dendrites. The error bars represent the s.e.m. and in some cases are smaller than the symbols used for the mean. n numbers are provided in the figure panels, which for panels a and b are regions of interest (ROIs) and for c and d are the numbers of cells.

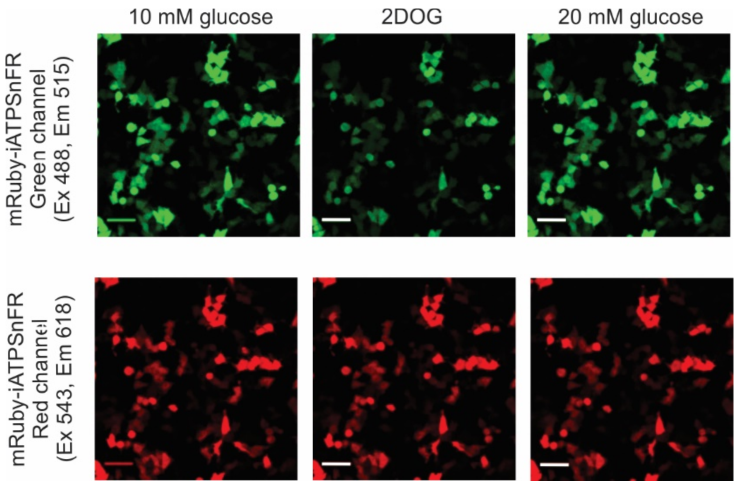

Supplementary Figure 5 Representative images for mRuby-iATPSnFR$^{1.0}$ expressed in HEK-293 cells and subjected to 2DOG (glycolysis inhibitor).

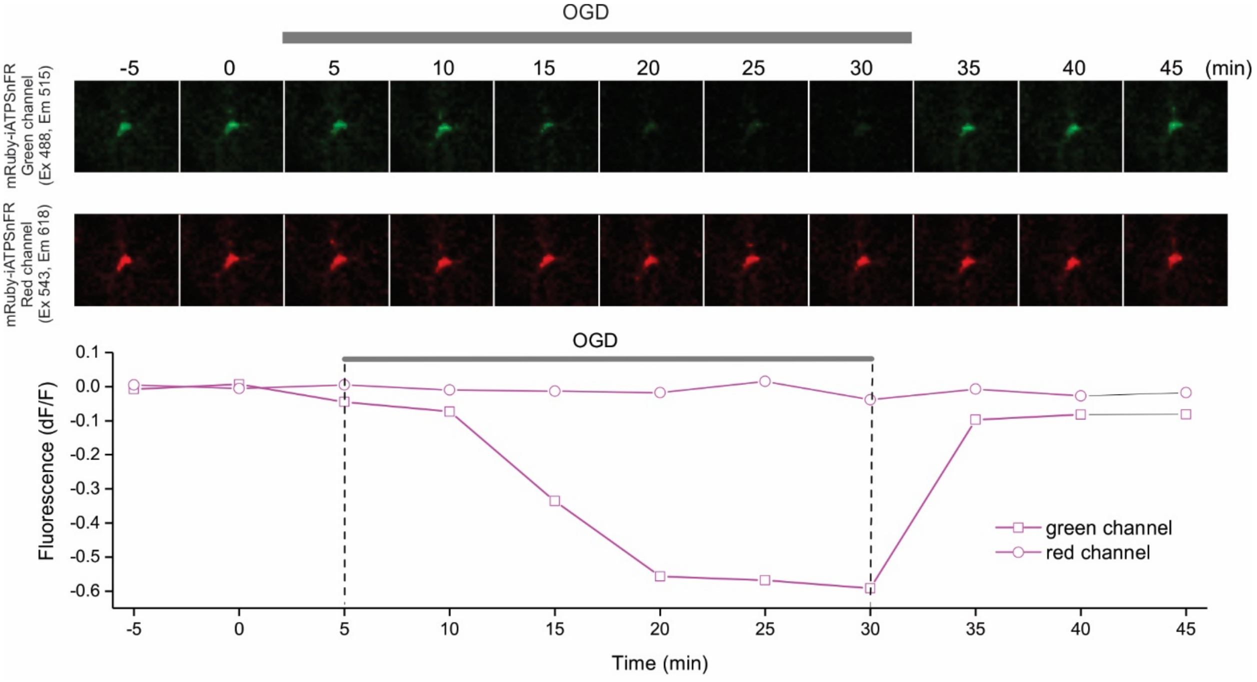

Supplementary Figure 6 Representative traces and images of an astrocyte expressing mRuby-iATPSnFR$^{1.0}$ in hippocampal brain slices following *in vivo* delivery with AAV2/5. Oxygen-glucose deprivation caused clear decreases in iATPSnFR$^{1.0}$ fluorescence, but left mRuby fluorescence intact.

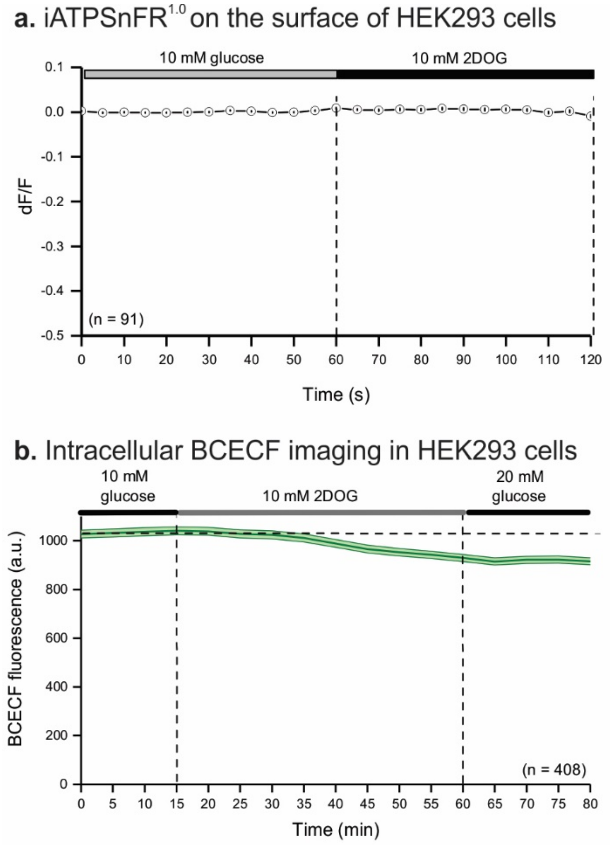

Supplementary Figure 7 Control experiments for iATPSnFR$^{1.0}$. (a) Cell surface iATPSnFR$^{1.0}$ did not respond to 10 mM 2DOG. Hence, 2DOG does not affect the optical properties of iATPSnFR$^{1.0}$ directly. **(b)** The pH indicator dye BCECF was used to image pH level changes in HEK293 cells before, during and after application of 10 mM 2DOG. A 10% drop in fluorescence, corresponding to around a 0.1 pH unit acidification, was detected. This occurred slowly and was not reversible after washout of 2DOG. The error bars represent the s.e.m. and in some cases are smaller than the symbols used for the mean. n numbers are provided in the figure panels.

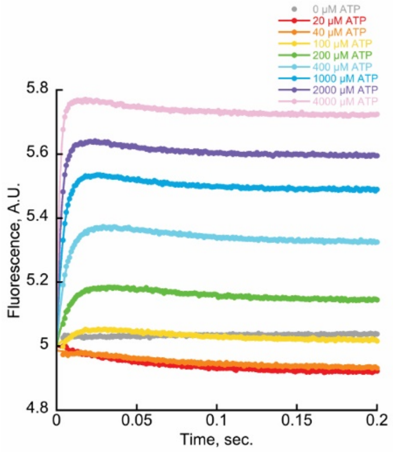

Supplementary Figure 8 Stopped-flow kinetics for iATPSnFR$^{1.0}$ measured in purified protein (from bacterial expression).

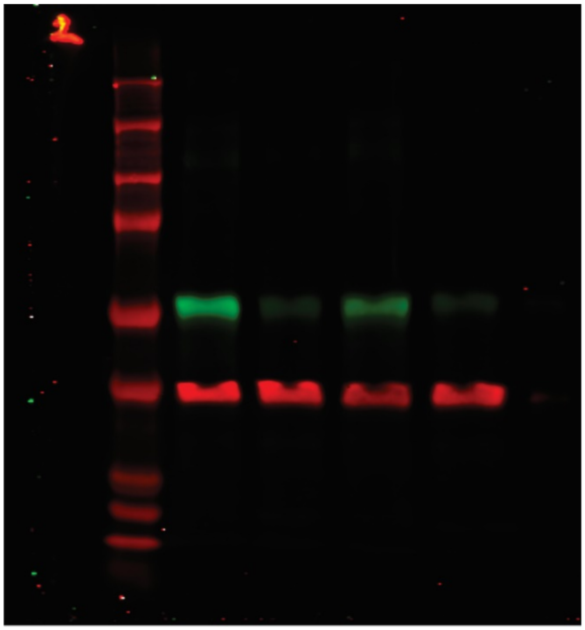

Supplementary Figure 9 Full uncropped gel to accompany Figure 3f of the main manuscript. The molecular weight markers are 250, 148, 98, 64, 50, 36, 22, 16, 6, 4 KDa. The first two lanes are shown on the main Figure 3f. Lanes 3 and 4 and the same experiment but from a different batch run on the same gel.

Supplementary Table 1 Comparison of known genetically encoded ATP sensors based on the use of fluorescent proteins.

	Sensor name	Type	Optical property	dF/F (dR/R)	Kd	pKa	t_{on} (s^{-1})	t_{off} (s^{-1})	Reference
cytosolic	AT1.03	FRET	mseCFP-cpVenus	1.3	3.3 mM	<7	ND	0.098	[1]
	AT1.03-YEMK	FRET	mseCFP-cpVenus	ND	1.2 mM	ND	ND	ND	[1]
	AT3.10	FRET	mseCFP-cpVenus	~1	7.4 µM	ND	ND	ND	[1]
	AT3.10-GMK	FRET	mseCFP-cpVenus	ND	14 µM	ND	ND	ND	[1]
	AT1.03-NL	FRET	mseCFP-cpVenus	0.99	1.77 mM	<7	ND	0.029	[2]
	GO-ATeam1	FRET	cpGFP-mKO	~1.2	7.1 mM	<6.3	ND	0.13	[3]
	GO-ATeam2	FRET	cpGFP-mKO	~2	2.3 mM	ND	ND	ND	[3]
	EAF-ATeam	FRET	GFP-YFP	ND	<10 µM	ND	ND	ND	[4]
	Perceval	cpmVenus	490 nm, ~515 nm	~2	0.04 µM	ND	ND	ND	[5]
	Perceval-HR	cpmVenus	420 nm, ~515 nm 490 nm	~7	3.4 µM	ND	ND	0.5	[6]
	MaLionG	Citrine	565 nm, 585 nm	3.9	1.1 mM	~6.7	ND	0.095	[7]
	MaLionR	mApple	505 nm, 522 nm	3.5	0.34 mM	~6.5	ND	0.016	[7]
	MaLionB	BFP	373 nm, 446 nm	0.9	0.46 mM	4.9	ND	0.015	[7]
	Queen-7 µM	cpGFP	400 nm, 513 nm 494 nm	~4	7 µM	<7	ND	ND	[8]
	Queen-2 mM	cpGFP	400 nm, 513 nm 494 nm	~3.5	2 mM	ND	ND	ND	[8]
	iATPSnFR$^{1.0}$	cpSFGFP	490 nm, 512 nm	2.4	120 µM	6.5	ND	ND	This study
	iATPSnFR$^{1.1}$	cpSFGFP	490 nm, 512 nm	1.9	50 µM	ND	ND	ND	This study
Cell surface	ecAT3.10	FRET	mseCFP-cpVenus	0.27	12 µM	ND	<0.0042	<0.01	[9]
	iATPSnFR$^{1.0}$	cpSFGFP	490 nm, 512 nm	1.0	350 µM	6.5	0.47	0.58	This study
	iATPSnFR$^{1.1}$	cpSFGFP	490 nm, 512 nm	0.9	140 µM	ND	1.1	0.58	This study

Key to table: ND means not determined. dR/R refers to the measurements with FRET based sensors and indicates a change (delta) in the FRET ratio, R upon ATP binding at maximal concentrations. The equivalent value for the single-wavelength sensors is dF/F. For the FRET-based sensors, the "optical property" indicates the donor and acceptor FPs. For the single-wavelength sensors, the "optical property" lists the excitation and emission wavelengths. As can be seen, the kinetics were not reported for most of the sensors, but from the available data the fastest ATP sensors are iATPSnFR$^{1.0}$ and iATPSnFR$^{1.1}$, which we characterized in this study.

Plasmid name	Expresses in	Vector	Addgene ID
HHM-iATPSnFR$^{1.0}$	Bacteria	HHM modified pRSET	102546
HHM-iATPSnFR$^{1.1}$	Bacteria	HHM modified pRSET	102547
pm-iATPSnFR$^{1.0}$	Mammalian cells	pDisplay	102548
pm-iATPSnFR$^{1.1}$	Mammalian cells	pDisplay	102549
cyto-iATPSnFR$^{1.0}$	Mammalian cells	modified pDisplay	102550
cyto-iATPSnFR$^{1.0}$-mScarlet	Mammalian cells	modified pDisplay	
cyto-iATPSnFR$^{1.0}$-P2A-mScarlet	Mammalian cells	modified pDisplay	
cyto-Ruby3-iATPSnFR$^{1.0}$	Mammalian cells	modified pDisplay	102551
cyto-LSSmOrange-iATP	Mammalian cells	modified pDisplay	
GfaABC$_1$D-pm-iATPSnFR$^{1.0}$	Mouse astrocytes	pZac	102552
GfaABC$_1$D-pm-iATPSnFR$^{1.1}$	Mouse astrocytes	pZac	
GfaABC$_1$D-cyto-iATPSnFR$^{1.0}$	Mouse astrocytes	pZac	102553
GfaABC$_1$D-cyto-iATPSnFR$^{1.0}$-mScarlet	Mouse astrocytes	pZac	
GfaABC$_1$D-cyto-Ruby3-iATPSnFR$^{1.0}$	Mouse astrocytes	pZac	102554
GfaABC$_1$D-cyto-LSSmOrange-iATPSnFR$^{1.0}$	Mouse astrocytes	pZac	
GfaABC$_1$D-cyto-iATPSnFR$^{1.0}$-P2A-mScarlet	Mouse astrocytes	pZac	
Synapsin-pm-iATPSnFR$^{1.0}$	Mouse neurons	pSynapsin	102555
Synapsin-pm-iATPSnFR$^{1.1}$	Mouse neurons	pSynapsin	
Synapsin-cyto-iATPSnFR$^{1.0}$	Mouse neurons	pSynapsin	102556
Synapsin-cyto-iATPSnFR$^{1.0}$-mScarlet	Mouse neurons	pSynapsin	
Synapsin-cyto-Ruby3-iATPSnFR$^{1.0}$	Mouse neurons	pSynapsin	102557
Synapsin-cyto-LSSmOrange-iATPSnFR$^{1.0}$	Mouse neurons	pSynapsin	
Synapsin-cyto-iATPSnFR$^{1.0}$-P2A-mScarlet	Mouse neurons	pSynapsin	

Supplementary Table 2 Details of the new plasmids generated in this study along with the Addgene IDs.

References

1 Imamura, H. *et al.* Visualization of ATP levels inside single living cells with fluorescence resonance energy transfer-based genetically encoded indicators. *Proc Natl Acad Sci U S A* **106**, 15651-15656 (2009).

2 Tsuyama, T. *et al.* In vivo fluorescent adenosine 5'-triphosphate (ATP) imaging of Drosophila melanogaster and Caenorhabditis elegans by using a genetically encoded fluorescent ATP biosensor optimized for low temperatures. *Anal Chem* **85**, 7889-7896 (2013).

3 Nakano, M., Imamura, H., Nagai, T. & Noji, H. Ca^{2+} regulation of mitochondrial ATP synthesis visualized at the single cell level. *ACS Chem Biol* **6**, 709-715 (2011).

4 Zadran, S. *et al.* Enhanced-acceptor fluorescence-based single cell ATP biosensor monitors ATP in heterogeneous cancer populations in real time. *Biotechnol Lett* **35**, 175-180 (2013).

5 Berg, J., Hung, Y. P. & Yellen, G. A genetically encoded fluorescent reporter of ATP:ADP ratio. *Nat Methods* **6**, 161-166 (2009).

6 Tantama, M., Martínez-François, J. R., Mongeon, R. & Yellen, G. Imaging energy status in live cells with a fluorescent biosensor of the intracellular ATP-to-ADP ratio. *Nat Commun* **4:2550. doi: 10.1038/ncomms3550.** (2013).

7 Arai, S. *et al.* RGB-Color Intensiometric Indicators to Visualize Spatiotemporal Dynamics of ATP in Single Cells. *Angew Chem Int Ed Engl* **57**, 10873-10878 (2018).

8 Yaginuma, H. *et al.* Diversity in ATP concentrations in a single bacterial cell population revealed by quantitative single-cell imaging. *Sci Rep* (2014).

9 Conley, J. M., Radhakrishnan, S., Valentino, S. A. & Tantama, M. Imaging extracellular ATP with a genetically-encoded, ratiometric fluorescent sensor. *PLoS One* **Nov 9;12(11):e0187481. doi: 10.1371/journal.pone.0187481** (2017).
