# Supplementary material for: A genetically encoded single-wavelength sensor for imaging cytosolic and cell surface ATP
Source: Nat Commun. 2019 Feb 12;10:711. doi: 10.1038/s41467-019-08441-5 (PMC6372613; doi:10.1038/s41467-019-08441-5)
Supplement: Supplementary file 2 — Description of Additional Supplementary Files [file 41467_2019_8441_MOESM2_ESM.docx]

**Description of Additional Supplementary Files**

File Name: Supplementary Movie 1

Description: Representative movie for ATP response for cell surface iATPSnFR^1.0^. The addition and washout of 100 µM and 1 mM ATP were conducted at 30-second intervals. We averaged the first 25 frames of images to obtain the baseline fluorescence image (F0). Then all the images were pseudocolored by F/F0 (calibration bar ranges from 0–2) and exported to JPEG format with a play speed of 7 fps. Scale bars, 10 µm.

File Name: Supplementary Movie 2

Description: Representative movie for ATP response for cell surface iATPSnFR^1.1^. The addition and washout of 100 µM and 1 mM ATP were conducted at 30-second intervals. We averaged the first 25 frames of images to obtain the baseline fluorescence image (F0). Then all the images were pseudocolored by F/F0 (calibration bar ranges from 0–2) and exported to JPEG format with a play speed of 7 fps. Scale bars, 10 µm.
