# Supplementary material for: A genetically encoded single-wavelength sensor for imaging cytosolic and cell surface ATP
Source: Nat Commun. 2019 Feb 12;10:711. doi: 10.1038/s41467-019-08441-5 (PMC6372613; doi:10.1038/s41467-019-08441-5)
Supplement: Supplementary file 5 — Reporting Summary [file 41467_2019_8441_MOESM5_ESM.pdf]

nature research

Corresponding author(s): Baljit S. Khakh
Last updated by author(s): Dec 24, 2018

Reporting Summary

Nature Research wishes to improve the reproducibility of the work that we publish. This form provides structure for consistency and transparency in reporting. For further information on Nature Research policies, see Authors & Referees and the Editorial Policy Checklist.

Statistics

For all statistical analyses, confirm that the following items are present in the figure legend, table legend, main text, or Methods section.

n/a	Confirmed	
☐	☒	The exact sample size (*n*) for each experimental group/condition, given as a discrete number and unit of measurement
☐	☒	A statement on whether measurements were taken from distinct samples or whether the same sample was measured repeatedly
☐	☒	The statistical test(s) used AND whether they are one- or two-sided<br>*Only common tests should be described solely by name; describe more complex techniques in the Methods section.*
☐	☒	A description of all covariates tested
☐	☒	A description of any assumptions or corrections, such as tests of normality and adjustment for multiple comparisons
☐	☒	A full description of the statistical parameters including central tendency (e.g. means) or other basic estimates (e.g. regression coefficient) AND variation (e.g. standard deviation) or associated estimates of uncertainty (e.g. confidence intervals)
☐	☒	For null hypothesis testing, the test statistic (e.g. *F*, *t*, *r*) with confidence intervals, effect sizes, degrees of freedom and *P* value noted<br>*Give P values as exact values whenever suitable.*
☒	☐	For Bayesian analysis, information on the choice of priors and Markov chain Monte Carlo settings
☒	☐	For hierarchical and complex designs, identification of the appropriate level for tests and full reporting of outcomes
☒	☐	Estimates of effect sizes (e.g. Cohen's *d*, Pearson's *r*), indicating how they were calculated

Our web collection on statistics for biologists contains articles on many of the points above.

Software and code

Policy information about availability of computer code

Data collection	TillVision Software (Till Photonics), Fluoview 1000 software (Olympus), iControl 1.10 (Tecan), NIS Elements V4.60 (Nikon)
Data analysis	Image J, FIJI, OriginPro 2016 64 bit, GraphPad Instat Version 3.10 32 bit, GECIquant plug in for Image J, Vector NTI (Thermo Fisher Scientific), Kaleidagraph 4.5 (Synergy Software)

For manuscripts utilizing custom algorithms or software that are central to the research but not yet described in published literature, software must be made available to editors/reviewers. We strongly encourage code deposition in a community repository (e.g. GitHub). See the Nature Research guidelines for submitting code & software for further information.

Data

Policy information about availability of data

All manuscripts must include a data availability statement. This statement should provide the following information, where applicable:
- Accession codes, unique identifiers, or web links for publicly available datasets
- A list of figures that have associated raw data
- A description of any restrictions on data availability

All the data are available upon request from the authors. All the newly generated plasmids have been deposited at Addgene with accession IDs that are listed in Supplementary Table 2.

Field-specific reporting

Please select the one below that is the best fit for your research. If you are not sure, read the appropriate sections before making your selection.

☒ Life sciences ☐ Behavioural & social sciences ☐ Ecological, evolutionary & environmental sciences

For a reference copy of the document with all sections, see nature.com/documents/nr-reporting-summary-flat.pdf

Life sciences study design

All studies must disclose on these points even when the disclosure is negative.

Sample size	No data points were excluded. Sample sizes were chosen based on past experience in the design and testing of biosensors for a variety of bioactive substances gathered over ~ 10 years and were chosen to give robust reproducible data.
Data exclusions	No data were excluded
Replication	All experiments were replicated. Full details are provided in the Data analysis section
Randomization	The allocation of mice to the various groups was random. Experiments with cell lines (e.g. HEK293) were not randomized as the cell lines are clonal, although multiple batches of the cell lines were used during the course of the work.
Blinding	Blinding was not used and was not possible as it is possible to see the transfected and/or infected cells by the experimenter. Such visual identification is necessary to identify the cells to image as neither the transfection or infection are 100% efficient.

Reporting for specific materials, systems and methods

We require information from authors about some types of materials, experimental systems and methods used in many studies. Here, indicate whether each material, system or method listed is relevant to your study. If you are not sure if a list item applies to your research, read the appropriate section before selecting a response.

Materials & experimental systems

n/a	Involved in the study	
☐	☒	Antibodies
☐	☒	Eukaryotic cell lines
☒	☐	Palaeontology
☐	☒	Animals and other organisms
☒	☐	Human research participants
☒	☐	Clinical data

Methods

n/a	Involved in the study	
☒	☐	ChIP-seq
☒	☐	Flow cytometry
☒	☐	MRI-based neuroimaging

Antibodies

Antibodies used	GAPDH anti-mouse (Thermofisher MA5-15738), GFP annti-rabbit (Invitrogen A11122), IRDye 680RD anti-mouse (Licor 926-68170), IRDye 800CW anti-rabbit (Licor 925-68024), S100β anti-rabbit (Abcam ab41548). NeuN anti-rabbit(Cell Signaling D3S3I), GFP anti-chicken (Abcam ab13970), goat anti-chicken IgG Alexa 488 (Invitrogen A11039), goat anti-rabbit Alexa 546 (Invitrogen A11010).
Validation	These are well characterized, standard antibodies that have been extensively used in our laboratories for several years with no problems. No specific validation of the antibodies was performed for these experiments and nothing untoward was noted with their use.

Eukaryotic cell lines

Policy information about cell lines

Cell line source(s)	HEK293 (ATCC, CRL-1573) and U373MG cells (Sigma-Aldrich, 08061901)
Authentication	These are standard cell lines used by us for several years. No additional authentication was performed for this specific study but they were periodically replaced by purchasing new vials from ATCC.
Mycoplasma contamination	They were not tested for mycoplasma at UCLA, but were tested for mycoplasma at Janelia.
Commonly misidentified lines (See ICLAC register)	HEK (has different genetic identity). We used HEK293 cells to be consistent with past work

Animals and other organisms

Policy information about studies involving animals; ARRIVE guidelines recommended for reporting animal research

Laboratory animals	C57BL/6NTac
Wild animals	N/A
Field-collected samples	N/A
Ethics oversight	All animal experiments were conducted in accordance with the National Institute of Health Guide for the Care and Use of Laboratory Animals and were approved by the Chancellor's Animal Research Committee at the University of California, Los Angeles

Note that full information on the approval of the study protocol must also be provided in the manuscript.
